# The Intrauterine and Nursing Period Is a Window of Susceptibility for Development of Obesity and Intestinal Tumorigenesis by a High Fat Diet in *Min/+* Mice as Adults

**DOI:** 10.1155/2015/624023

**Published:** 2015-03-19

**Authors:** Ha Thi Ngo, Ragna Bogen Hetland, Inger-Lise Steffensen

**Affiliations:** Department of Food, Water and Cosmetics, Division of Environmental Medicine, Norwegian Institute of Public Health, P.O. Box 4404 Nydalen, 0403 Oslo, Norway

## Abstract

We studied how obesogenic conditions during various life periods affected obesity and intestinal tumorigenesis in adult C57BL/6J-*Min* (multiple intestinal neoplasia)/+ mice. The mice were given a 10% fat diet throughout life (negative control) or a 45% fat diet *in utero*, during nursing, during both *in utero* and nursing, during adult life, or during their whole life-span, and terminated at 11 weeks for tumorigenesis (*Min/+*) or 23 weeks for obesogenic effect (wild-type). Body weight at 11 weeks was increased after a 45% fat diet during nursing, during both *in utero* and nursing, and throughout life, but had normalized at 23 weeks. In the glucose tolerance test, the early exposure to a 45% fat diet *in utero*, during nursing, or during both *in utero* and nursing, did not affect blood glucose, whereas a 45% fat diet given to adults or throughout life did. However, a 45% fat diet during nursing or during *in utero* and nursing increased the number of small intestinal tumors. So did exposures to a 45% fat diet in adult life or throughout life, but without increasing the tumor numbers further. The intrauterine and nursing period is a window of susceptibility for dietary fat-induced obesity and intestinal tumor development.

## 1. Introduction

Obesity is defined as an excess accumulation of adipose tissue. The rate of obesity has more than doubled over the past 20 years in most OECD countries [1]. More than half of the adult population are overweight (with body mass index (BMI) 25–30 kg/m^2^) or obese (with BMI ≥30 kg/m^2^), and about 18% of both genders are obese. Rates of overweight and obesity among children are also increasing; average reported overweight rates (including obesity) increased from 13% in 2001-2002 to 15% in 2009-2010 for 15-year-olds (based on age- and gender-specific cut-off points for BMI) [1]. Maternal obesity during pregnancy is also a serious health issue with a prevalence of obese adult women close to 30% in many of the OECD countries [2].

A parallel increase in overweight/obesity and many forms of cancer has been observed in most countries around the world in the past two to three decades. Cancer is now the second leading cause of mortality in the OECD countries [1]. In Norway, colon cancer is the second most prevalent cancer for women, after breast cancer, and the third most prevalent cancer for men, after prostrate and lung cancer [3]. Overweight and/or obesity are associated with increased risk, incidence, mortality, or poor prognosis for many types of cancer, including colon cancer [4–7]. Body fatness and abdominal fatness are both evaluated as convincing increasing risks of colorectal cancer [8, 9]. Obesity may be a contributing risk factor for increased susceptibility to environmental contaminants causing cancer.

The rapid rise of obesity is suggested to be driven mainly by environmental factors. Although it has been much focus on the role of the current diet whether as an obese child or adult, recent insights have also stressed the importance of nutrition during very early life in the development of metabolic disorders. The phenotype of an individual can be driven by* in utero *and early postnatal environmental conditions, determined by the nutritional status of the mother [10]. This has given rise to the perception of “developmental programming” and the concept “developmental origins of health and disease” (DOHaD). It is proposed that conditions present during a critical window of development can lead to permanent programmed alterations in physiological systems and adverse outcomes later in life [10, 11].

The “fetal origins of adult disease hypothesis” was originally put forward by David Barker and colleagues, which stated that environmental factors, especially nutrition, act in early life to program the risks for early onset of diseases such as hypertension, diabetes, coronary heart disease, metabolic disorders, and mental illnesses in adult life and premature death [10–12]. Although the initial fetal origins hypothesis was primarily concerned with undernutrition and malnutrition, recent epidemiological and animals studies have begun to examine the effects of overnutrition during critical periods of fetal development and the offspring's subsequent risk of developing the same chronic diseases associated with fetal growth restriction [13].

Maternal obesity is associated with numerous pregnancy-related complications and risks for both mother and child [14–16]. In addition to infertility, the mothers may have increased risk from obesity for hypertensive disorders, coagulopathies, gestational diabetes mellitus, respiratory complications, pre-eclampsia, thromboembolism, and so forth, in addition to miscarriage. The fetus has increased risk of large-for-gestational-age size, congenital malformations or perinatal mortality [14–16].

In this study, we have examined overnutrition, in the form of a high fat diet, during various periods of life in relation to the end points body weight and intestinal tumorigenesis in the mice as adults, using the C57BL/6J-*Min/+ (multiple intestinal neoplasia)* mouse as the experimental animal model. In addition, the wild-type (+/+) siblings were used to examine the effects on body weight and organ weights in older mice.

The* Min/+* mouse is heterozygous for a germline nonsense mutation in the tumor suppressor gene* adenomatous polyposis coli (Apc)* leading to a truncated nonfunctional APC protein, and therefore develop numerous spontaneous intestinal tumors [17, 18].* Apc* is a key component in the Wnt signaling pathway [19, 20]. The* Min* mouse is a model for the inherited disorder familial adenomatous polyposis (FAP), as well as for sporadic colorectal cancer, in humans [21–23], and develops multiple adenomas in the small intestine and to a lesser degree in the colon.

In addition to the effects on spontaneous intestinal tumors caused by the inherited mutated* Apc* gene in the* Min/+* mice, the effect of obesity was also examined on tumors induced by the environmental (dietary) factor formed during cooking of meat and fish, the mutagenic, genotoxic and carcinogenic heterocyclic amine 2-amino-1-methyl-6-phenylimidazo[4,5-*b*]pyridine (PhIP) [24]. Previously, we have reported that PhIP increased intestinal tumorigenesis in adult C57BL/6J-*Min/+* mice [25], and that the* Min/+* mice were much more susceptible to PhIP if exposed neonatally [26, 27] than as young adults [25, 27]. Blood glucose levels were measured and a glucose tolerance test (GTT) was performed to study the hypothesis of disrupted blood glucose regulation as a link between obesity and intestinal tumorigenesis [28, 29]. The hormone leptin, which regulates food intake and energy expenditure, as well as having effects on immunity, including inflammation, and reproduction [30, 31], was measured in serum from the mice.

In this study, we have examined during which periods of life does exposure to obesogenic conditions in the form of a high fat diet have the most effect on body weight and susceptibility to disease, that is, intestinal tumorigenesis, as adults.

## 2. Materials and Methods

### 2.1. Mice

Female C57BL/6J-*Apc* ^*+/+*^ (wild-type) mice were mated with C57BL/6J-*Apc* ^*Min/+*^ males, using proven breeders having had a litter on a regular breeding diet (2018 Teklad Global 18% Protein Rodent Diet from Harlan Industries Inc., Indianapolis, IN, USA) before the experimental litters on special diets with 10% or 45% fat (described below). Both females and males were bred at the Norwegian Institute of Public Health, Oslo, Norway. C57BL/6J-*Apc* ^*Min/+*^ males were originally purchased from the Jackson Laboratory (Bar Harbour, ME, USA). To minimize the genetic drift from the colony at the Jackson Laboratory, both females and males in the breeding stock at our institute have been replaced regularly. Homozygous mutant* Apc* ^*Min/Min*^ (*Apc* ^−/−^) mice die during the embryonal stages [32]; therefore, only two genotypes were obtainable from these crosses. The* Min* mutation was propagated through males to avoid interference with pregnancy from any anemia caused by the intestinal adenomas in females [17].

Genotyping of the offspring for the* Apc* gene was performed with allele-specific polymerase chain reaction (PCR) using DNA extracted from ~2 mm^2^ samples obtained by ear puncture for identification of individual mice at weaning, as described previously [33].

The mice were housed in air flow IVC racks (Innovive Inc., San Diego, CA, USA) in 100% PET plastic disposable cages on Nestpak Aspen 4HK bedding (Datesand Ltd., Manchester, UK) in a room with 12-h light/dark cycle, and controlled humidity (55 ± 5%) and temperature (20–24°C). Diet and water were given* ad libitum*.

The experiment reported in this paper was performed in conformity with the laws and regulations for animal experiments in Norway and was approved by the National Experimental Animal Board in Norway.

### 2.2. Experimental Diets

**Figure 1:**
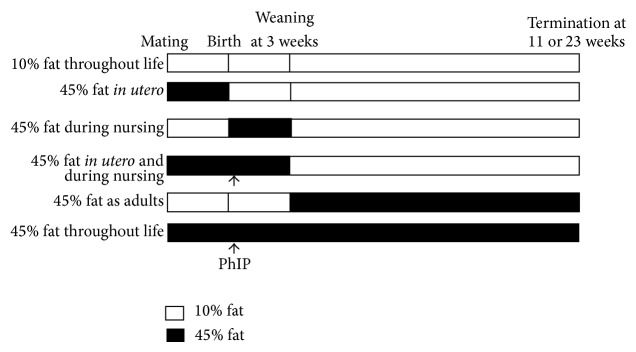
Experimental design. The mice were exposed to a 45% fat diet for combinations of three periods in life; (1)* in utero*, via the dams, (2) from birth to weaning, via milk during nursing, or (3) from weaning at 3 weeks to termination at 11 weeks of age (for* Min/+* mice) or 23 weeks (for wild-type mice), to determine the most susceptible exposure period for development of obesity and intestinal tumorigenesis as adults. The effects of a 45% fat diet were studied on spontaneous tumorigenesis induced by the inherited mutation in the* Apc* gene and on tumors induced by the the food mutagen and carcinogen 2-amino-1-methyl-6-phenylimidazo[4,5-*b*]pyridine (PhIP). The mice in two experimental groups (marked with arrows) were given one s.c. injection of 25 mg/kg body weight of PhIP on days 3–6 after birth. In total, eight experimental groups were included in this experiment; a 10% fat diet throughout life as a negative control (10+10+10), a 45% fat diet* in utero* (45+10+10), a 45% fat diet during the nursing period (10+45+10), a 45% fat diet both* in utero* and during nursing (45+45+10), exposed to PhIP or not, a 45% fat diet as adults (10+10+45), or a 45% fat diet throughout life (45+45+45), exposed to PhIP or not.

Diets of purified ingredients from Research Diets Inc. (New Brunswick, NJ, USA) were used. The D12451 diet, containing 20%, 35%, and 45% of kcal from protein, carbohydrates, and fat, respectively, was used as a high fat diet. The D12450H diet, containing 20%, 70%, and 10% of kcal from protein, carbohydrates, and fat, respectively, was used as a matching control low fat diet. The amount of sucrose was 17% of the calories in both diets. The high fat diet had 4.73 kcal/g, whereas the low fat diet had 3.85 kcal/g; that is, the high fat diet contained 22.9% more kcal per gram diet. In order to avoid that the dietary treatment was unevenly spread out in time, we gave the first dam 10% fat diet, the second dam 45% fat diet, the third dam 10% fat diet, the forth dam 45% fat diet, and so on. Similarly, the litters of offspring were given either of the two diets after birth every other time and after weaning every other time until the necessary numbers in all experimental dietary groups were obtained (Figure 1). The number of litters (given in parentheses) in each treatment group was 10% fat diet throughout life (17), 45% fat diet* in utero* (17), 45% fat diet during the nursing period (14), 45% fat diet* in utero* and during the nursing period (19), 45% fat diet as adults (18), and 45% fat diet throughout life (21). For the groups also given PhIP, the number of litters was 45% fat diet* in utero* and during the nursing period (17) and 45% fat diet throughout life (17). The number of mice in each treatment group is given in the figures and tables for the various end points.

### 2.3. Dietary Carcinogen

2-Amino-1-methyl-6-phenylimidazo[4,5-*b*]pyridine (PhIP) hydrochloride (CAS number 105650-23-5), catalogue number 163-15951, of >99% purity, was purchased from Wako Chemicals GmbH, Neuss, Germany. PhIP-HCl was dissolved in distilled water, and the pH was adjusted to approximately 4.0.

### 2.4. Experimental Treatment of Mice

The mice were exposed to a 10% fat control diet or a 45% fat diet during combinations of three periods of life; (1)* in utero*, via the dams, (2) from birth to weaning, via milk during nursing, and (3) from weaning at 3 weeks to termination at 11 weeks of age (the* Min/+* mice) or 23 weeks (the wild-type mice), to determine the most susceptible exposure period for development of obesity and intestinal tumorigenesis as adults. The effects of a high fat diet were studied on spontaneous tumorigenesis induced by the inherited mutation in the* Apc* gene, and on tumors induced by the the food mutagen and carcinogen PhIP. The mice in two experimental groups were given one s.c. injection of 25 mg/kg body weight of PhIP on days 3–6 after birth. This dose of PhIP was chosen to give a suitable number of tumors above the spontaneous level based on previous experience [34]. In total, eight treatment groups were included in this experiment (Figure 1). The number of mice (*n*) in each treatment group is given for each end point in the figure legends and tables.

Blood was sampled by cardiac puncture under anesthesia with ZRF cocktail (containing 3.3 mg zolazepam, 3.3 mg tiletamine, 0.5 mg xylazine and 2.6 *μ*g fentanyl per mL 0.9% NaCl) into Microvette serum/clot activator tubes (Sarstedt AS, Ski, Norway), and serum was obtained for analysis of the hormone leptin. Thereafter, the mice were sacrificed by cervical dislocation.

### 2.5. Recording of Body Weight and Feed Intake

Body weight of the dams was recorded at mating and weekly during the pregnancy and lactation periods. Body weight of the offspring was registered on day 3-4 after birth and thereafter weekly from weaning until termination of the* Min/+* mice at 11 weeks of age, before onset of noticeable anemia caused by their tumors. The wild-type mice were terminated at 23 weeks of age, to study the effects on body weight and organ weights at older age. However, the body weight of the wild-type mice was also evaluated at 11 weeks of age, for comparison with the* Min/+* mice terminated at 11 weeks. Body weight data were evaluated in three ways; as body weight at a specific age (at 11 weeks for the* Min/+* mice and at 23 weeks for the wild-type mice), terminal body mass index (BMI) (also at 11 weeks for the* Min/+* mice and at 23 weeks for the wild-type mice), and as body weight development expressed as area under the curve (AUC). The AUC was calculated for the offspring from age 3-4 days to week 11 (*Min/+* and wild-type mice), and from week 12 to 23 (wild-type mice), as well as for the dams from mating to the end of pregnancy, using the trapezoidal rule in SigmaPlot 12.3 (Systat Software Inc., San Jose, CA, USA). Nasoanal length was also recorded at termination to calculate BMI as body weight/nasoanal length^2^ (in g/cm^2^). Feed intake was monitored by weighing feed in and out of the cages weekly for the dams during the pregnancy and lactation periods, and for the pups from weaning until termination.

### 2.6. Scoring of Small Intestinal and Colonic Tumors

Colon and small intestine were removed separately, rinsed in ice-cold phosphate buffered saline (PBS) and slit open along the longitudinal axis. Intestinal tissues were then spread flat between sheets of filter paper, and fixed for at least 48 h in 10% neutral buffered formalin prior to staining with 0.2% methylene blue (Sigma-Aldrich Norway AS, Oslo, Norway). Number, diameter and localization of tumors in small intestine and colon were scored by transillumination in an inverse light microscope at a magnification of ×20. The scoring was done in order of consecutive mouse numbers unaware of their treatment. Diameters of tumors were scored with an eyepiece graticule. Tumor position along the intestines was registered in cm from the stomach. For each experimental group, incidence of tumors (number of mice with tumors/number of mice in the group), tumor number (mean number of tumors/mouse ± SD) and tumor diameter in mm (mean of all tumors in all mice in the group ± SD) were calculated, for small intestine and colon separately. In addition, the size of the tumors was illustrated by curves of distributions of tumor size classes (of 0.25 mm tumor diameter intervals), calculated as mean number of tumors in each tumor size class for each treatment group. These curves were used to illustrate the effects of a 45% fat diet for various periods, which were calculated by subtracting the mean number of tumors in mice exposed to the control diet with 10% fat throughout life from the mean number of tumors in mice exposed to a 45% fat diet for various periods (Figure 10(a)). It was also done to illustrate the effect of PhIP on tumor size, by subtracting the mean tumor numbers in the corresponding dietary groups not exposed to PhIP from the mean number of tumors in the PhIP-treated groups (Figure 10(b)).

### 2.7. Absolute and Relative Organ Weights

The liver and spleen were dissected and weighed at termination, and the data are presented as absolute weight (in gram), or as relative weight (in %) calculated as absolute weight/body weight × 100.

### 2.8. Blood Glucose Measurements and Glucose Tolerance Test (GTT)

Nonfasted blood glucose was measured with a glucometer (FreeStyle Freedom Lite, Abbott Diabetes Care, Inc., Alameda, CA, USA) in all the mice by puncture of the saphenous vein in the hind leg at two time points: at age 6 and 11 weeks (*Min/+* mice) and at age 6 and 23 weeks (wild-type mice).

The glucose tolerance test (GTT) was performed on a subset of mice from each treatment group when they were 10 weeks old. The mice were fasted for 6 h from approximately 9 a.m. to 3 p.m. before i.p. injection of 2 g/kg body weight D-(+) glucose (Sigma-Aldrich, Norway, AS, Oslo). Blood glucose was measured 5 min before and 15, 30, 60 and 120 min after injection of glucose. The AUC was calculated from −5 to 120 min with the trapezoidal rule using Sigmaplot 12.3.

When readings were above 27.8 mmol/L and displaying HIGH on the glucometer, this value was used in the data analysis. This was not registered in any of the nonfasted blood glucose samples. In GTT, this occurred only for one* Min/+* male given a 45% fat diet as adult at the 15 min time point, and for one* Min/+* female given a 45% fat diet throughout life and PhIP, at 60 min. No samples in either end point had glucose readings below 1.1 mmol/L and showing LOW in the glucometer.

### 2.9. Leptin ELISA

The hormone leptin was measured in serum obtained from the mice at sacrifice. An ELISA kit (catalogue number MBS455345) from MyBioSource Inc. (San Diego, CA, USA) was used according to the manufacturer's instructions. Optical density (OD) was measured at 450 nm on a BioTek microplate reader (BioTek Instruments Inc., Winooski, VT, USA). Concentrations were calculated from standard curves on each plate. All samples were diluted 1 : 20 in PBS, pH 7.1. The limit of detection was 0.06 ng leptin/mL.

### 2.10. Statistical Analyses

The data are presented as mean ± SD and were analysed using SigmaPlot 12.3. The incidence of colonic tumors was analysed by Fischer exact test (two-tailed probability). For evaluation of all other data, analysis of variance (ANOVA) was used with an appropriate multiple comparison procedure. When testing the influence of a single factor, one-way ANOVA with the Holm-Sidak test for multiple comparisons was used for parametric data or the Kruskal-Wallis ANOVA on ranks with Dunn's test for multiple comparisons was used for nonparametric data. When testing the influence of two or three factors together the data were analysed by two- or three-way ANOVA, respectively, with the Holm-Sidak test for multiple comparisons. A *P* value of < 0.05 was considered statistically significant.

## 3. Results

### 3.1. The Dams' Age When Mated

There were no statistically significant differences between the mean age at mating for the dams given the various dietary combinations, being 121 days (10+10+10, *n* = 17), 108 days (45+10+10, *n* = 17), 120 days (10+45+10, *n* = 14), 122 days (45+45+10, *n* = 19), 114 days (10+10+45, *n* = 18), 104 days (45+45+45, *n* = 21), 112 days (45+45+10PhIP, *n* = 17), and 105 days (45+45+45PhIP, *n* = 17). There was a statistically significant difference (*P* = 0.046) between the mean age at mating of all the dams given a 10% fat versus a 45% fat diet, with mean age of 118 days (range 88–170 days) for the dams on the 10% fat diet and 110 days (range 82–186) on the 45% fat diet. However, this small difference most likely has no biological significance.

### 3.2. Breeding Efficiency on the Various Diets

There were no statistically significant differences in the resulting mean number of pups per litter in the various experimental dietary groups, being 6.1 (10+10+10, *n* = 17), 6.4 (45+10+10, *n* = 17), 7.4 (10+45+10, *n* = 14), 5.7 (45+45+10, *n* = 19), 6.9 (10+10+45, *n* = 18), 6.9 (45+45+45, *n* = 21), 6.2 (45+45+10PhIP, *n* = 17) and 6.2 (45+45+45PhIP, *n* = 17). Likewise, there were no statistically significant differences in the mean number of pups per litter between all litters given a 10% fat diet (6.8, *n* = 49) or a 45% fat diet (6.3, *n* = 91) during pregnancy.

### 3.3. Feed Intake of the Dams during Pregnancy

**Figure 2:**
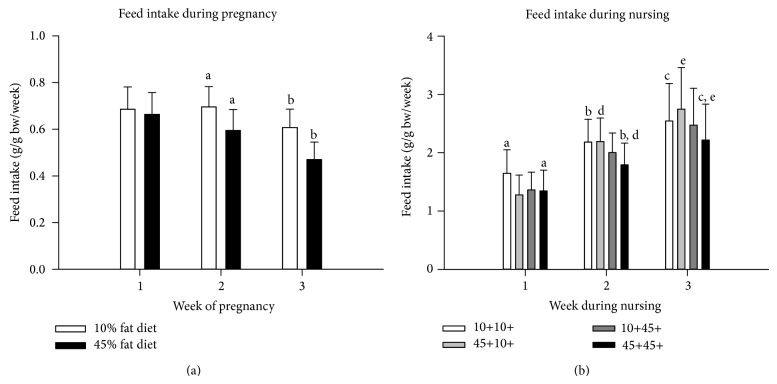
The feed intake of the dams during (a) pregnancy, that is, from mating to the end of week 3 of pregnancy (*n* = 37–119), and (b) the three week lactation period (*n* = 12–69), recorded as gram diet per gram body weight per week (mean ± SD). During pregnancy, the dams were given either a 10% fat (white columns) or a 45% fat diet (black columns). The data for the lactation period were stratified according to the four different combinations of the 10% fat or 45% fat diet during pregnancy and the 10% fat or 45% fat diets during the lactation period; 10+10+ (white columns), 45+10+ (light grey columns), 10+45+ (dark grey columns), 45+45+ (black columns), as explained in the legend to Figure 1. (a) ^a, b^Significantly higher with a 10% fat diet versus a 45% fat diet within the same week. (b) ^a, b, c^Significantly higher with a 10% fat diet during both pregnancy and nursing periods versus a 45% fat diet in the same periods within the same week. (b) ^d, e^Significantly higher with a 45% fat diet during pregnancy and a 10% fat diet during nursing versus a 45% fat diet in both periods within the same week.

The feed intake of the mice dams was recorded as gram feed per gram body weight per week for each of the three weeks of pregnancy (Figure 2(a)). The dams had a significantly higher feed intake per gram body weight in both week 1 and 2 compared with week 3, of both the 10% fat and 45% fat diets (*P* < 0.001 for all comparisons), and in week 1 compared with week 2 for the 45% fat diet (*P* < 0.001). The intake of feed per gram body weight was higher for the 10% fat diet than the 45% fat diet for all three weeks together, and for weeks 2 and 3 separately (*P* < 0.001 for all comparisons).

### 3.4. Feed Intake of the Dams during Nursing

The feed intake of the mice dams was recorded as gram feed per gram body weight per week for each of the three weeks of nursing (Figure 2(b)). The dams in all dietary groups had a significantly higher feed intake in both week 2 and 3 compared with week 1, and in week 3 compared with week 2, in all mice and in each experimental dietary group separately (*P* values were <0.001 to 0.012). The dams given a 45% fat diet during pregnancy and lactation periods had significantly lower feed intake than the dams given a 10% fat diet in both periods, in all time periods together and in weeks 1, 2 and 3, separately (*P* values were <0.001 to 0.018). The feed intake of the 45% fat diet compared with the 10% fat diet fat diet was 18.3, 17.9 and 12.8% lower in week 1, 2 and 3, respectively. The dams given a 45% fat diet during both pregnancy and the lactation period also had significantly lower feed intake than the dams given a 45% fat diet during pregnancy and a 10% fat diet during lactation, in all time periods together and in weeks 2 and 3, separately (*P* values were <0.001 to 0.010).

### 3.5. Feed Intake of the* Min/+* and Wild-Type Offspring Aged 4 to 11 Weeks

**Figure 3:**
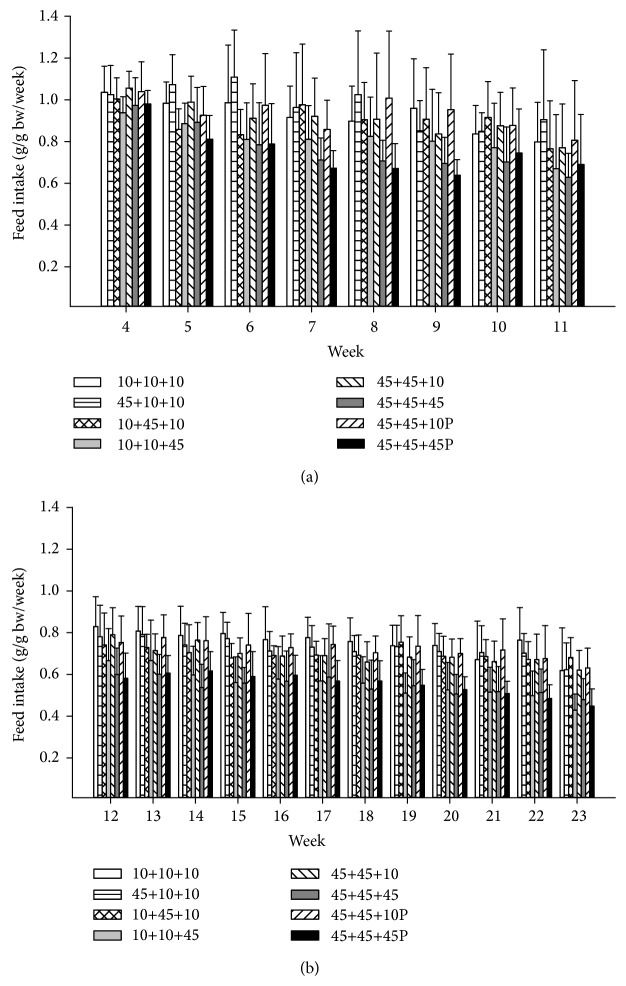
The feed intake of the mice offspring after weaning was recorded as gram diet per gram body weight per week (mean ± SD) for each experimental group (*n* = 10–27), shown for (a) female* Min/+* and wild-type mice combined, from 4 to 11 weeks of age, and for (b) wild-type males, from 12 to 23 weeks of age. Experimental dietary groups: 10+10+10 (open white columns), 45+10+10 (horizontally striped columns), 10+45+10 (cross-hatched columns), 10+10+45 (light grey columns), 45+45+10 (left upwards diagonally striped columns), 45+45+45 (dark grey columns), 45+45+10 + PhIP (right upwards diagonally striped columns), 45+45+45 + PhIP (filled black columns), as explained in the legend to Figure 1.

The feed intake of the mice offspring (*Min/+* and wild-type combined) after weaning was recorded as gram feed per gram body weight per week for each experimental group from week 4 to 11 for females (Figure 3(a)) and males (data not shown) separately. In general, females had a higher feed intake than males on gram body weight basis (*P* < 0.001), as has been found in our previous experiments (see [33], Ngo et al., 2014; unpublished results). This was seen in all treatment groups separately (*P* = 0.004 in the negative control group and the group given 45% fat diet throughout life and PhIP, and *P* < 0.001 for the rest of the groups), and in all weeks separately (*P* = 0.007 at week 4 and *P* < 0.001 for the other weeks), except week 5 which did not reach significance. There was a general decrease in feed intake per gram body weight each week compared with the following week (Figure 3(a)), which was statistically significant (*P* values were 0.018 to < 0.001) except for weeks 7–10.

The mice given a 45% fat diet either as adults (for 8 weeks) or throughout life (for 11 weeks), with or without PhIP, had significantly lower feed intake per gram body weight per week than the other dietary groups receiving a 10% fat diet throughout life (for 11 weeks), or a 45% fat diet for shorter time, that is, only* in utero* (for 3 weeks), only during nursing (for 3 weeks) or during* in utero* and nursing (for 6 weeks), with or without PhIP (*P* values from 0.002 to < 0.001). PhIP did not affect the feed intake.

### 3.6. Feed Intake of the Wild-Type Offspring Aged 12 to 23 Weeks

The feed intake of the wild-type mice offspring was recorded as gram feed per gram body weight per week for each experimental group from week 12 to 23 for females (data not shown) and males (Figure 3(b)), separately. In general, wild-type females had a higher feed intake than males on gram body weight basis (*P* < 0.001), which was also seen in all treatment groups separately and in all weeks separately (*P* < 0.001 for all comparisons). There was generally a higher feed intake per gram body weight for the earlier weeks compared with the later weeks (Figure 3(b)), which was statistically significant in females for weeks 12, 14, 15 and 17 compared with each of weeks 20–23, and for week 12 also compared with weeks 18 and 19, and for week 15 also compared with week 19. Feed intake in week 16 was significantly higher compared with weeks 21–23, and feed intake in week 13 and weeks 18–22 was significantly higher than in week 23 only (*P* values were 0.048 to < 0.001). In males, there was a significantly higher feed intake in weeks 12 and 13 compared with each of weeks 20–23, and for weeks 14–22 compared with week 23 (*P* values were 0.041 to < 0.001).

As observed for the* Min/+* and wild-type mice at weeks 4–11, the wild-type mice of both genders at weeks 12–23 given a 45% fat diet either as adults (for 8 weeks) or throughout life (for 11 weeks), with or without PhIP, had significantly lower feed intake per gram body weight per week than the other dietary groups receiving a 10% fat diet throughout life (for 11 weeks), or a 45% fat diet for shorter time, that is, only* in utero* (for 3 weeks), only during nursing (for 3 weeks) or during* in utero* and nursing (for 6 weeks), with or without PhIP (*P* < 0.001 for all comparisons). PhIP did not affect the feed intake.

### 3.7. Body Weight of the Dams during Pregnancy in Gram or as AUC

**Figure 4:**
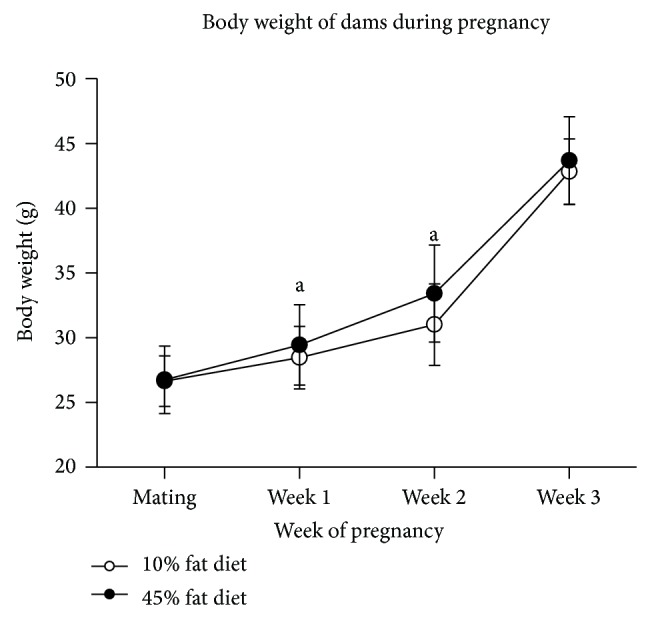
Body weight development (in gram) of dams during pregnancy, that is, from mating to the end of week three of pregnancy (mean ± SD). The dams were given either a 10% fat (open circles) or a 45% fat (filled circles) diet in this period. *n* = 41–123. ^a^Significantly higher with a 45% fat diet versus a 10% fat diet in the same week.

The increase in body weight in gram for the dams on either a 10% fat or a 45% fat diet from mating until the end of pregnancy is shown in Figure 4. The body weight was significantly increased each week compared with the previous week for dams on both 10% fat and 45% fat diets (*P* values were < 0.001 or 0.002 for all comparisons). The body weight was significantly higher in the dams on a 45% fat diet compared with a 10% fat diet at week 1 (3.3%, *P* = 0.028) and week 2 (7.2%, *P* < 0.001) of pregnancy, but not at mating (0.4%) and at week 3 at the end of pregnancy (2.0%).

Also when calculating the increase in body weight as AUC from mating until the end of pregnancy, the dams on a 45% fat diet had significantly higher AUC than the dams on a 10% fat diet (*P* = 0.018) (data not shown).

### 3.8. Body Weight in* Min/+* and Wild-Type Mice Offspring in Gram or as AUC from Day 3-4 to Week 11

**Figure 5:**
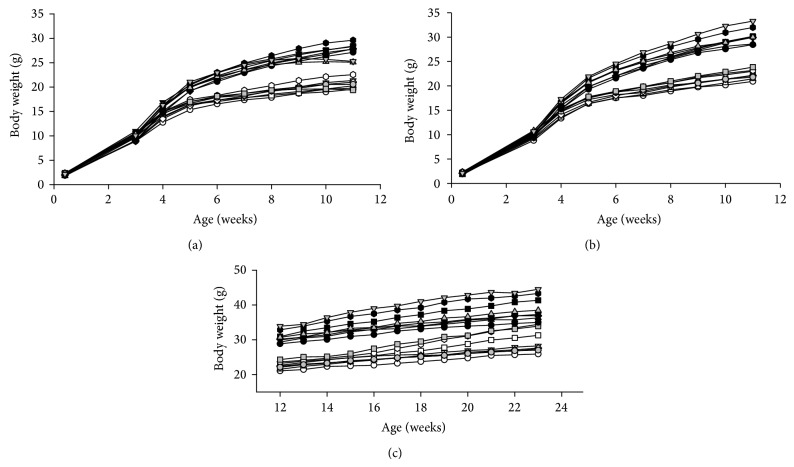
Body weight development (in gram) of female and male mice of all treatment groups is illustrated for (a)* Min/+* and (b) wild-type mice from age 3-4 days to 11 weeks, and for (c) wild-type mice from age 12 to 23 weeks. The open white symbols are for untreated female mice; 10+10+10 (○), 45+10+10 (△), 10+45+10 (*▽*), 45+45+10 (□), 10+10+45 (◊), 45+45+45 (open hexagon), and the same filled black symbols are for untreated male mice. The PhIP-treated groups are marked with grey symbols; 45+45+10 PhIP (○) and 45+45+45 PhIP (□) in females, and 45+45+10 PhIP (△), 45+45+45 PhIP (*▽*) in males. The experimental groups are as explained in the legend to Figure 1. *n* = 20–46.

**Figure 6:**
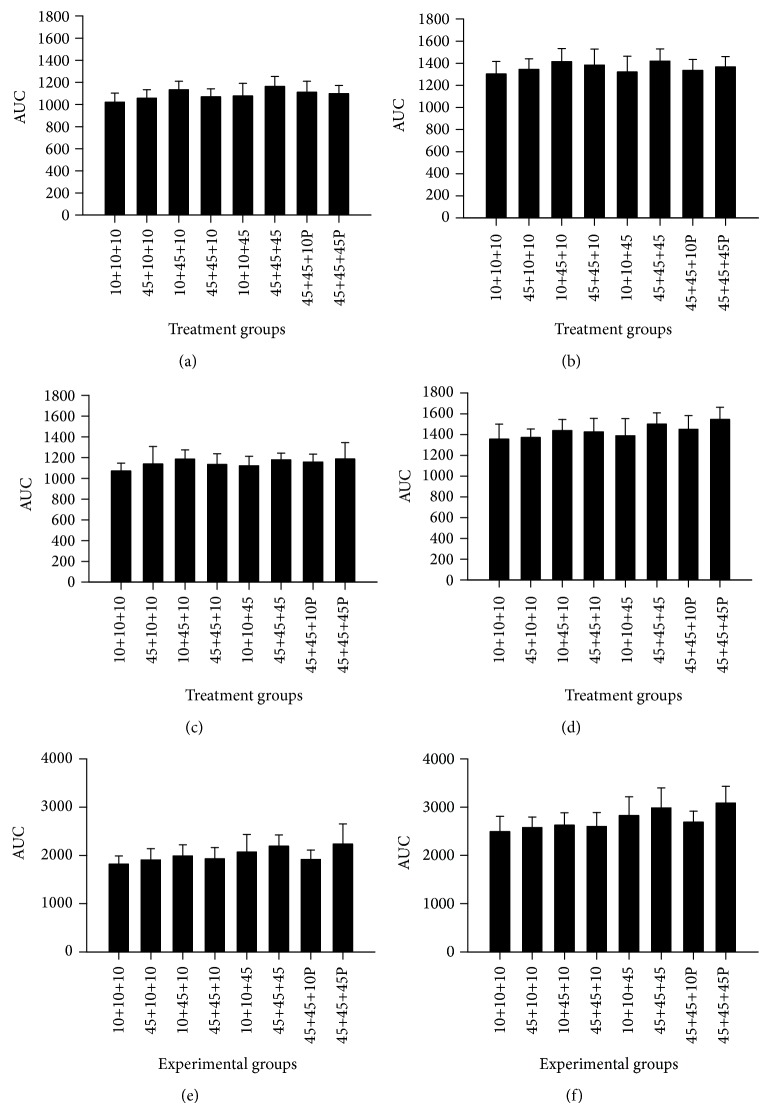
Body weight development as AUC (arbitrary units, mean ± SD) from age 3-4 days to 11 weeks for (a)* Min/+* females, (b)* Min/+* males, (c) wild-type females and (d) wild-type males, and from age 12 to 23 weeks for (e) wild-type females and (f) wild-type males. The experimental groups are as explained in the legend to Figure 1. *n* = 20–46.

Body weight development (in gram) for both female and male* Min/+* (Figure 5(a)) and wild-type (Figure 5(b)) mice of all treatment groups is shown from age 3-4 days to 11 weeks. The body weight development over time of the mice offspring was evaluated statistically as area under the curve (AUC) from day 3-4 to week 11 for* Min/+* (Figures 6(a) and 6(b)) and wild-type (Figures 6(c) and 6(d)) mice for each dietary group. The* Min/+* mice had a lower AUC compared with the wild-type mice in both females and males, and in mice both with and without PhIP treatment (*P* < 0.001 for all comparisons). Both* Min/+* and wild-type male mice had larger AUC than females (*P* < 0.001 both comparisons), which was apparent in all dietary groups (*P* < 0.001 for all comparisons).* Min/+* mice exposed to PhIP had lower body weight than mice not exposed to PhIP (*P* = 0.027), but PhIP did not affect the body weight in the wild-type mice.

In both* Min/+* and wild-type mice of both genders, although slightly increased, exposure to a 45% fat diet* in utero* did not significantly increase the body weight as AUC compared with the negative control group given a 10% fat diet throughout life. Exposure to a 45% fat diet only during the nursing period significantly increased AUC compared with the negative control group (*P* ≤ 0.009), except in the subgroup male wild-type mice. However, exposure to a 45% fat diet both* in utero* and during nursing significantly increased AUC compared with the negative control group when evaluating both genotypes and genders together (*P* < 0.001), but only in the subgroup of males for* Min/+* and wild-type mice together (*P* = 0.010), whereas each genotype and gender separately did not reach significance. The exposure to a 45% fat diet during* in utero* and nursing did not increase AUC further compared with a 45% diet only during nursing. Thus, the effect of a 45% fat diet during the nursing period is more efficient in increasing the body weight than the exposure* in utero*.

The exposure to a 45% fat diet as adults did not increase AUC compared with the control group or any of the other exposure groups. The exposure to a 45% fat diet during nursing actually gave a significantly higher AUC than exposure as adults* in Min/+* male mice (*P* = 0.011). In both* Min/+* and wild-type mice of both genders, the exposure to a 45% fat diet throughout the whole life increased AUC compared with the negative control group (*P* < 0.001), and this was also the case for all subgroups;* Min/+* females (*P* < 0.001),* Min/+* males (*P* = 0.001), wild-type females (*P* = 0.006) and wild-type males (*P* < 0.001). Exposure to a 45% fat diet throughout life also increased AUC compared with the group given a 45% fat diet only* in utero* (*P* < 0.001), which was seen in female (*P* = 0.024) and male (*P* < 0.001) mice of both genotypes combined, and also in the subgroups female* Min/+* mice (*P* = 0.012) and male wild-type mice (*P* < 0.001). Exposure to a 45% fat diet throughout life also increased AUC compared with exposure both* in utero* and during nursing (*P* < 0.001), which was seen in the female (*P* = 0.024) and male (*P* = 0.030) mice of both genotypes combined, and in the subgroup female* Min/+* mice (*P* = 0.022). The comparison of a 45% fat diet throughout life with the group given a 45% fat diet only during nursing did not reach significance. However, AUC was significantly higher after exposure to a 45% fat diet throughout life compared with a 45% fat diet given only as adults (*P* < 0.001), which was seen in female (*P* = 0.004) and male (*P* < 0.001) mice of both genotypes combined, and also in the subgroups female (*P* = 0.024) and male (*P* = 0.003)* Min/+* mice, and male (*P* < 0.001), but not female, wild-type mice.

Within the PhIP-exposed* Min/*+ mice, there was no significant difference in AUC between the exposure to a 45% fat diet during* in utero* and nursing compared with throughout life.

### 3.9. Body Weight in Wild-Type Mice Offspring in Gram or as AUC from Week 12 to 23

The* Min/+* mice were terminated at 11 weeks of age, before negative health effects of their tumors become apparent. The wild-type mice were not terminated until 23 weeks of age to study the impact of the early life exposure to a 45% fat diet in older mice. The body weight in gram of the wild-type mice from age 12 to 23 weeks is illustrated in Figure 5(c). The body weight development of the wild-type mice offspring was evaluated statistically as AUC from week 12 to week 23 for each dietary group (Figures 6(e) and 6(f)). The male wild-type mice were significantly heavier than the females also at this age (*P* < 0.001). Similar to the wild-type mice from day 3-4 to week 11, there was no difference in AUC between wild-type mice given PhIP or left untreated from week 12 to 23.

In the wild-type mice of both genders, although slightly increased, exposure to the 45% fat diet only* in utero* did not significantly increase the body weight as AUC from week 12 to 23 compared with the negative control group given a 10% fat diet throughout life. The same was the case with exposure to a 45% fat diet only during nursing, or during both* in utero* and nursing. In wild-type mice, the exposure to a 45% fat diet as adults or throughout life increased AUC from week 12 to 23 compared with the negative control group, both in females (*P* = 0.033 and *P* < 0.001) and in males (*P* < 0.001 for both comparisons), respectively.

Exposure to a 45% fat diet as adults also increased AUC compared with the group given a 45% fat diet only* in utero*, and both* in utero* and during nursing, in males (*P* = 0.0013 and *P* = 0.046, resp.), but not in females. Exposure to a 45% fat diet throughout life also increased AUC compared with exposure* in utero*, and* in utero* and during nursing, in females (*P* = 0.008 and *P* = 0.009, resp.), and in males (*P* < 0.001 for both comparisons). Exposure to a 45% fat diet throughout life also increased AUC compared with exposure only during the nursing period in males (*P* < 0.001), but not in females. The AUC results were not significantly different between a 45% fat diet as adults or throughout life in either gender.

Thus, the effects of an early exposure to a 45% fat diet both* in utero* and during nursing, or only during nursing, on AUC observed at age 3-4 days to 11 weeks, were no longer present when the wild-type mice had reached the age of 12 to 23 weeks.

### 3.10. Body Weight in* Min/+* and Wild-Type Mice Offspring at a Specific Age (Week 11)

Body weight at 11 weeks of age was evaluated for both* Min/+* (Figure 5(a)) and wild-type mice (Figure 5(b)) of both genders. The* Min/+* mice had significantly lower terminal body weight than the wild-type mice (*P* < 0.001). This was observed in the subgroups untreated mice (*P* = 0.002), PhIP-treated mice (*P* < 0.001), and in females (*P* < 0.001) and males (*P* < 0.001), separately. Male mice had significantly higher body weight at termination at 11 weeks compared with the female mice, in* Min/+* mice, in wild-type mice, in untreated and PhIP-treated mice, and in all dietary groups (*P* < 0.001 for all comparisons).

Based on all mice and* Min/+* mice separately, mice exposed to PhIP had a significantly lower terminal body weight compared with the untreated mice (*P* < 0.001 for both comparisons), but this was not seen in wild-type mice separately. PhIP affected body weight in both female (*P* = 0.033) and male* Min/+* mice (*P* < 0.001), separately. This was also seen in the subgroups given 45% fat diet* in utero* and during nursing (*P* = 0.019) and throughout life (*P* < 0.001). Apparently, the tumor burden in the* Min/+* mice, which is increased further with PhIP exposure, affects their body weight negatively, before overt signs of anemia and other negative health effects were observed. The same results were also observed in a previous study with this mouse model (Ngo et al., 2014; unpublished results).

Based on both* Min/+* and wild-type mice, a 45% fat diet given* in utero* did not increase terminal body weight compared with the 10% control diet, whereas exposure during the nursing period only (*P* < 0.001) or during both* in utero* and nursing period (*P* = 0.032) did. In* Min*/+ or wild-type mice separately, a 45% fat diet given* in utero*, or during both* in utero* and nursing period, did not increase the body weight, whereas exposure during only the nursing period did (*P* = 0.045 for* Min/+* mice, and *P* = 0.033 for wild-type mice). In female and male mice separately, none of these comparisons reached significance.

Based on both* Min/+* and wild-type mice, exposure to a 45% fat diet only as adults increased the body weight compared with a 10% fat diet (*P* = 0.012), but not compared with exposure to a 45% fat diet* in utero*, during nursing, or during both* in utero* and nursing, whereas none of these comparisons reached significance in* Min/+* and wild-type mice separately, or in female and male mice separately.

A 45% fat diet given throughout life to* Min/+* and wild-type mice combined, or both genotypes separately, gave a significantly higher body weight compared with the negative control group given a 10% fat diet throughout life (*P* < 0.001 for all comparisons). Exposure to a 45% fat diet throughout life to the* Min/+* and wild-type mice combined also had significantly higher body weight compared with all the other dietary groups (*P* values from 0.013 to < 0.01). This was also seen in* Min/+* and wild-type mice separately (*P* values from 0.037 to < 0.01), except that in these cases the comparison with the mice given a 45% fat diet during nursing did not reach significance. A 45% fat diet throughout life gave a higher body weight in the subgroup female* Min/+* mice compared with a 45% fat diet* in utero* (*P* = 0.035) and* in utero* and during nursing (*P* = 0.002), and in male wild-type mice compared with a 45% fat diet* in utero* (*P* < 0.001),* in utero* and during nursing (*P* = 0.046), and as adults (*P* = 0.019).

In the PhIP-treated* Min/+* mice, a 45% fat diet given throughout life did not give significantly higher body weight compared with exposure to a 45% fat diet only* in utero* or during nursing, as it did in the untreated* Min/+* mice (*P* < 0.001).

The 45% fat diet throughout life gave significantly higher body weight than the exposure to a 45% fat diet only in adult life (*P* < 0.001, *P* = 0.004 and *P* = 0.0022, in all mice, and* Min/+* mice and wild-type mice, resp.).

### 3.11. Body Weight in Wild-Type Mice Offspring at a Specific Age (Week 23)

The body weight of the wild-type mice from age 12 to 23 weeks is illustrated in Figure 5(c). Similar to the AUC results in wild-type mice from day 3-4 to week 11 and from week 12 to 23, there was no difference in terminal body weight at week 23 between mice given PhIP or left untreated. In these wild-type mice of both genders, although slightly increased, exposure to a 45% fat diet only* in utero* did not significantly increase terminal body weight at week 23 compared with the negative control group given a 10% fat diet throughout life. The same was the case with exposure to a 45% fat diet only during nursing, or both* in utero* and during nursing. The exposure to a 45% fat diet as adults or throughout life increased terminal body weight at week 23 compared with the negative control group, in both females and males separately (*P* < 0.001 for all comparisons).

Exposure to a 45% fat diet as adults increased terminal body weight compared with the group given 45% fat diet* in utero*, and both* in utero* and during nursing, in females (*P* = 0.043 and *P* = 0.040, resp.), and in males (*P* < 0.001 and *P* = 0.006, resp.), and compared with a 45% fat diet only during nursing in males (*P* = 0.024). Exposure to a 45% fat diet throughout life also increased terminal body weight compared with exposure* in utero*, during nursing, and both* in utero* and during nursing, in both females and males (*P* < 0.001 for all comparisons). The terminal body weight at 23 weeks was not significantly different between mice given a 45% fat diet as adults or throughout life in either gender.

Thus, the effects of an early exposure to a 45% fat diet observed during the* in utero* and nursing periods, or only during nursing, on terminal body weight at age 11 weeks, were no longer present when the wild-type mice had reached the age of 23 weeks.

### 3.12. Terminal BMI in* Min/+* Mice Offspring at Week 11

When terminating the* Min/+* mice at 11 weeks of age, terminal body weight and nasoanal length were recorded and BMI was calculated as body weight divided by the square of the nasoanal length (in g/cm^2^) (data not shown). The male* Min/+* mice had significantly higher BMI at termination at 11 weeks compared with the females, and this was observed in all experimental dietary groups (*P* < 0.001 for all comparisons). Mice exposed to PhIP had a significantly lower BMI compared with the untreated* Min/+* mice (*P* < 0.001), and this was seen in both mice given a 45% fat diet during* in utero* and nursing, and throughout life (*P* = 0.009 and *P* < 0.001, resp.), consistent with the other body weight results (AUC for body weight development and body weight at a specific time point).

None of the exposures to a 45% fat diet early in life;* in utero*, during nursing, or both* in utero* and during nursing, increased BMI compared with the negative control mice given a 10% fat diet throughout life, in either gender. The same results were found with exposure to a 45% fat diet as adults. The BMI after a 45% fat diet as adults was not significantly different from after a 45% fat diet* in utero*, during nursing, or during both* in utero* and nursing. A 45% fat diet given throughout life increased BMI compared with the negative control group, and the mice given a 45% fat diet* in utero*, in both genders separately (*P* < 0.001 and *P* = 0.021 in females, respectively, and *P* = 0.013 and *P* = 0.032, in males, resp.). A 45% fat diet given throughout life also increased BMI compared with a 45% fat diet* in utero* and during nursing in females (*P* < 0.001), and compared with a 45% fat diet during nursing (*P* = 0.048) and compared with as adults (*P* = 0.011) in males.

As opposed to body weight development as AUC from day 3-4 to week 11 and terminal body weight at 11 weeks, the end point terminal BMI at 11 weeks did not demonstrate early life as a sensitive period for obesity from exposure to a high fat diet in the* Min/+* mice.

### 3.13. Terminal BMI in Wild-Type Mice Offspring at Week 23

The wild-type mice were terminated at week 23, and their BMI values were calculated (data not shown). The male wild-type mice had significantly higher BMI at termination at 23 weeks compared with the females, and this was observed in all dietary groups (*P* < 0.001 for all comparisons). At this time point, there was no longer any significant difference in BMI between mice exposed to PhIP or left untreated.

As found for* Min/+* mice at 11 weeks, none of the exposures of wild-type mice to a 45% fat diet early in life;* in utero*, during nursing, or both* in utero* and during nursing, increased BMI compared with in the negative control mice given a 10% fat diet throughout life, in either gender. A 45% fat diet as adults increased the BMI compared with the negative control group (*P* < 0.001, for both genders), and compared with exposure to a 45% fat diet given* in utero* (*P* = 0.014 and *P* = 0.003) and both* in utero* and during nursing (*P* = 0.042 and *P* = 0.040), in females and males, respectively, and compared with a 45% fat diet given during nursing in females (*P* = 0.047). A 45% fat diet given throughout life increased BMI compared with the negative control mice, the mice given a 45% fat diet* in utero*, during nursing, and both* in utero* and during nursing, in both genders (*P* < 0.001 for all comparisons). The terminal BMI at 23 weeks was not significantly difference between exposure to a 45% fat diet as adults or throughout life.

Within the PhIP-exposed* Min/+* mice, there was no significant difference in terminal BMI between the exposure to a 45% fat diet during the* in utero* and nursing period compared with throughout life, either in females or males.

As was found for* Min/+* mice at 11 weeks of age, the end point terminal BMI at 23 weeks did no longer demonstrate the sensitive period for obesity early in life from exposure to a high fat diet in the wild-type mice.

### 3.14. Blood Glucose Levels

**Figure 7:**
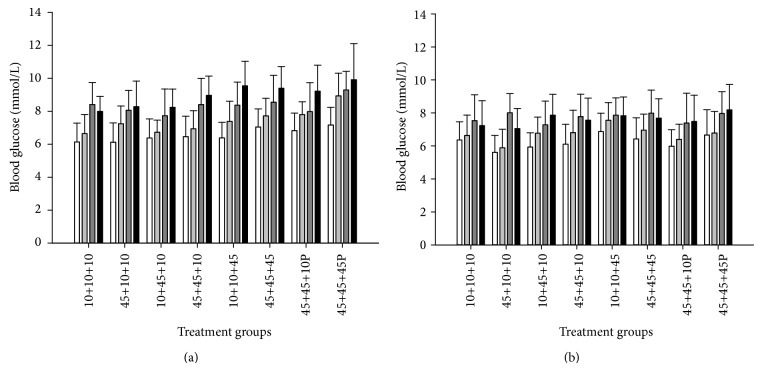
Nonfasted blood glucose levels (mmol/L, mean ± SD) for (a)* Min/+* mice; females at 6 and 11 weeks and males at 6 and 11 weeks, (b) wild-type mice; females at 6 and 23 weeks and males at 6 and 23 weeks, shown for both genotypes with columns in white, light grey, dark grey and black color, respectively. P = PhIP. The experimental groups are as explained in the legend to Figure 1. *n* = 9–46.

To test the hypothesis that obesity may affect intestinal tumorigenesis by disturbing the blood glucose regulation, blood glucose levels (nonfasted) were measured in all mice. This was done at weeks 6 and 11 in the* Min/+* mice (Figure 7(a)), and at weeks 6 and 23 in the wild-type mice (Figure 7(b)). When compared at 6 weeks, the* Min/+* mice had higher levels of blood glucose than the wild-type mice (*P* < 0.001), which was also found in our previous experiments (see [33], Ngo et al., 2014; unpublished results). The blood glucose levels were significantly higher in male compared with female* Min/+* mice, at both 6 and 11 weeks, and in wild-type mice, at both 6 and 23 weeks, and in mice treated with PhIP or not (*P* < 0.001 all comparisons). It was also seen in all dietary groups for both time points together (*P* < 0.001 for all comparisons). The blood glucose results are presented for females and males separately (Figure 7).

The blood glucose levels measured at week 11 was significantly higher than at week 6, for females (*P* < 0.001) and for males (*P* < 0.001), for PhIP-treated (*P* < 0.001) and for untreated (*P* = 0.018)* Min/+* mice. Blood glucose levels measured at week 23 were significantly higher than at week 6 for wild-type females (*P* < 0.001), whereas for males, the levels were higher at week 6 than week 23 (*P* = 0.036). Based on all values from* Min/+* mice at 6 and 11 weeks, the PhIP-treated mice had higher blood glucose levels than mice not treated with PhIP (*P* = 0.004), which was also observed in a previous experiment (Ngo et al., 2014; unpublished results), whereas this effect of PhIP was not significant for the wild-type mice at weeks 6 and 23.

Exposure to a 45% fat diet only* in utero*, during nursing, or both* in utero* and during nursing, did not significantly increase blood glucose levels compared with the negative control group given a 10% fat diet throughout life, neither in* Min/+* mice or wild-type mice at any time point.

Based on both genders at 6 and 11 weeks, a 45% fat diet given in adult life significantly increased the blood glucose levels compared with the control diet (*P* = 0.038) and exposure to a 45% fat diet during nursing (*P* = 0.009), but not compared with exposure to a 45% fat diet* in utero*, or both* in utero* and during nursing, in the* Min/+* mice. Based on both genders at 6 and 23 weeks, a 45% fat diet given in adult life significantly increased the blood glucose levels compared with the control diet (*P* = 0.006), exposure to a 45% fat diet* in utero* (*P* < 0.001) and during nursing (*P* = 0.033), but not compared with exposure to a 45% fat diet both* in utero* and during nursing, in the wild-type mice. Based on both 6 and 23 weeks, a 45% fat diet given in adult life to female wild-type mice separately significantly increased the blood glucose level compared with the control diet (*P* = 0.030), exposure to a 45% fat diet* in utero* (*P* < 0.001), during nursing (*P* = 0.019), and also compared with exposure to a 45% fat diet both* in utero* and during nursing (*P* = 0.018). In male wild-type mice separately, there were no statistically significant effects of a 45% fat diet given in adult life.

Based on both genders at 6 and 11 weeks, a 45% fat diet given throughout life significantly increased the blood glucose levels compared with the negative control group (*P* < 0.001), the group exposed to the 45% fat diet* in utero* (*P* = 0.001), and during nursing (*P* < 0.001), but the differences were not statistically significant compared with exposure to a 45% fat diet both* in utero* and during nursing, or as adults, in the* Min/+* mice. Based on both genders at 6 and 23 weeks, in the wild-type mice a 45% fat diet given throughout life significantly increased the blood glucose level compared with the group exposed to the 45% fat diet* in utero* (*P* < 0.001), but the differences were not statistically significant compared with the negative control group, exposure to a 45% fat diet during nursing, both* in utero* and during nursing, or as adults. Also based on both 6 and 11 weeks, in female wild-type mice separately a 45% fat diet given throughout life significantly increased the blood glucose levels compared with the group exposed to the 45% fat diet* in utero* (*P* = 0.001), whereas the comparisons with the other groups did not reach significance. In male wild-type mice separately, none of the various dietary groups were significantly different. At 6 weeks separately, none of the comparisons with the 45% fat dietary groups reached significance compared with the control group, and at 11 weeks separately, the only significant difference was between the 45% fat diet throughout life compared with the control group (*P* = 0.031).

A shorter exposure to a 45% fat diet early in life, that is,* in utero*, during nursing, or both* in utero* and during nursing, was apparently not able to affect the blood glucose levels, whereas a longer exposure to a 45% fat diet as adults or throughout life did increase the blood glucose levels. However, exposure to a 45% diet as adults, and not throughout life, increased blood glucose levels significantly more than exposure both* in utero* and during nursing only in the subgroup of female wild-type mice evaluated at 6 and 23 weeks (*P* = 0.018).

### 3.15. Glucose Tolerance Test (GTT)

**Figure 8:**
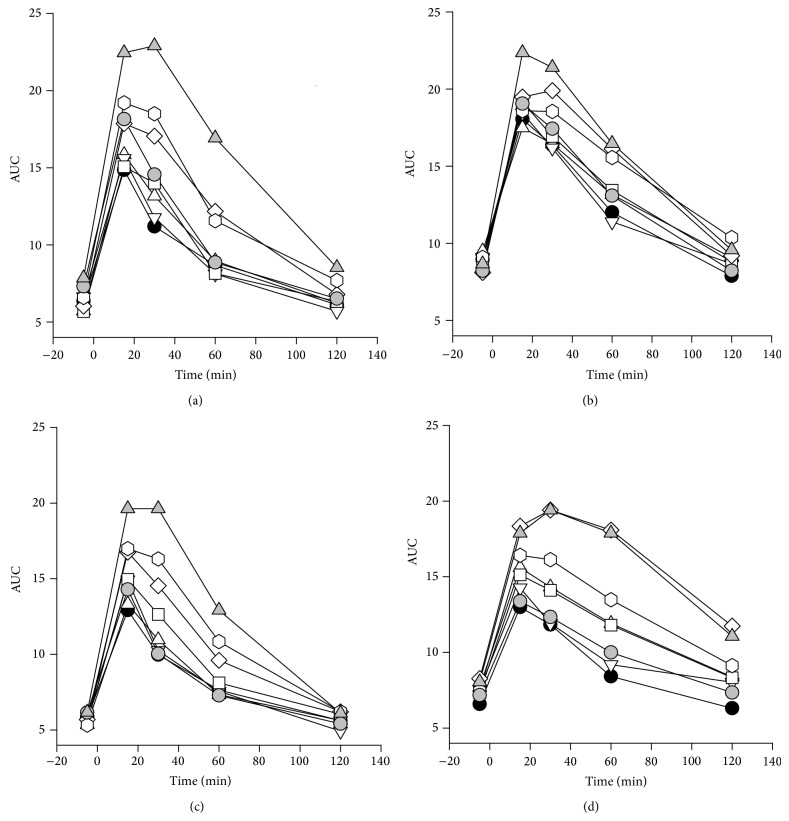
Mean fasted blood glucose levels at age 10 weeks as area under the curve (AUC) (arbitrary units) in the glucose tolerance test in (a) female and (b) male* Min/+* mice, and in (c) female and (d) male wild-type mice. The symbols for the untreated mice of both genders are: 10+10+10 (●), 45+10+10 (△), 10+45+10 (*▽*), 45+45+10 (□), 10+10+45 (◊), and 45+45+45 (open hexagon), and the symbols for the PhIP-treated mice are in grey color; 45+45+10 PhIP (○) and 45+45+45 PhIP (△). The experimental groups are as explained in the legend to Figure 1. *n* = 9–16.

To get a clearer picture of the effect on blood glucose regulation by the various dietary combinations, GTT was performed at 10 weeks on a subset of mice from each treatment group in a fasted state. A larger area under the curve (AUC) in the glucose tolerance test indicates that the mice have reduced ability to clear the injected glucose from the blood. As for the nonfasted blood glucose levels, significantly larger AUC was found in the* Min/+* mice (Figures 8(a) and 8(b)) compared with the wild-type mice (Figures 8(c) and 6(d)) (*P* < 0.001). And likewise, males had significantly higher AUC compared with females (*P* < 0.001), which is especially noticeable in the wild-type mice (Figures 8(c) and 6(d)).

Blood glucose AUC was higher in PhIP-exposed mice compared with mice not given PhIP after exposure to a 45% fat diet throughout life (*P* < 0.001), but not after exposure to a 45% fat diet* in utero* and during nursing (Figure 8).

The early exposure to a 45% diet* in utero*, during nursing, or during both* in utero* and nursing, did not differ from the control group given a 10% diet throughout life (Figure 8). The mice in the groups exposed to a 45% fat diet as adults or throughout life had significantly higher AUC than the negative control group (*P* < 0.001 for both comparisons), and the mice exposed to a 45% fat diet either during adult life or throughout life also had significantly higher AUC compared with all the other treatment groups, including the group given a 45% fat diet both* in utero* and during nursing (*P* = 0.002 or *P* < 0.001 for all comparisons) (Figure 8). The two dietary groups exposed to a 45% fat diet either during adult life or throughout life did not have significantly different AUC.

These GTT results essentially confirmed the results obtained by measuring blood glucose in a nonfasted state.

According to WHO [35], diagnostic criteria for humans with impaired glucose tolerance (IGT) are 7.8–11.1 mmol/L of glucose measured 2 h after an oral dose of 75 gram glucose, and levels above 11.1 mmol/L confirm diabetes. Regarding these levels also relevant for mice, we found IGT at the 2 h time point in the GTT in 8% and 23% (10+10+10), 5% and 69% (45+10+10), 0% and 68% (10+45+10), 27% and 60% (45+45+10), 13% and 82% (10+10+45), 23% and 70% (45+45+45), 10% and 50% (45+45+10 PhIP), and 32% and 92% (45+45+45 PhIP) of the female and male mice, respectively (treatment groups in parentheses). A diabetic level of glucose at the 2 h time point in the GTT was found in 0% and 0%, 0% and 7%, 0% and 5%, 5% and 5%, 0% and 27%, 0% and 22%, 0% and 0%, and 5% and 25% of the female and male mice in the same treatment groups as above, respectively.

### 3.16. Small Intestinal Tumors

**Figure 9:**
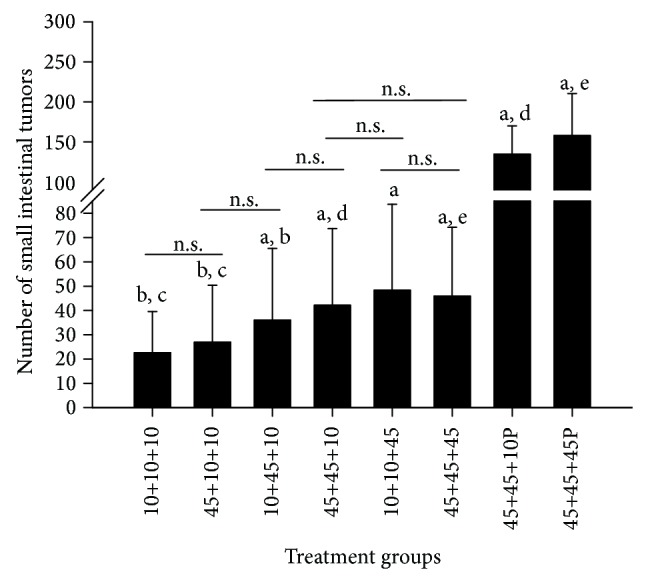
The number of small intestinal tumors for pooled male and female* Min/+* mice (mean ± SD). Two separate experimental groups receiving a 45% fat diet during* in utero* and nursing period or throughout life were injected with PhIP (marked P). The experimental groups are as explained in the legend to Figure 1. *n* = 44–65. ^a^Significantly different from the negative control group given a 10% fat diet throughout life. ^b^Significantly different from the group given a 45% fat diet throughout life. ^c^Significantly different from the group given a 45% fat diet* in utero* and during nursing. ^d^Significantly different with PhIP compared with no PhIP exposure in the groups given a 45% fat diet during* in utero* and nursing period. ^e^Significantly different with PhIP compared with no PhIP exposure in the groups given a 45% fat diet throughout life. n.s. = not significantly different.

All mice had small intestinal tumors (adenomas), independent of dietary or carcinogenic exposures, confirming 100% incidence of small intestinal tumors as is commonly found in the* Min/+* mice [25–27, 33]. The number of small intestinal tumors was not significantly different between the genders, thus the data for males and females are presented together (Figure 9). Although slightly increased, exposure to the 45% fat diet only* in utero* did not significantly increase the number of small intestinal tumors compared with the negative control group given a 10% fat diet throughout life. Exposure to a 45% fat diet only during the nursing period was significantly increased compared with the negative control group (*P* < 0.05). However, exposure to a 45% fat diet both* in utero* and during nursing, significantly increased the number of small intestinal tumors further, compared with the negative control group (*P* < 0.05), and compared with a 45% diet only* in utero* (*P* < 0.05), but not compared with only during nursing. Thus the effect of a 45% fat diet during the nursing period is more efficient in increasing the tumor number than the exposure* in utero*.

Both exposure to a 45% fat diet in adult life or throughout life gave a significant increase in small intestinal tumors compared with the negative control group, the group given a 45% fat diet* in utero*, and the group given a 45% fat diet only during nursing (*P* < 0.05 for all comparisons). However, exposure to a 45% fat diet in adult life or throughout life did not significantly increase the number of small intestinal tumors compared with the early exposure during both the* in utero* and nursing periods, showing the importance of early life exposure for intestinal tumor development by a high fat diet. Unexpectedly, the exposure to a 45% fat diet throughout life did not increase the number of small intestinal tumors compared with this exposure only during adult life.

To compare the effects of a 45% fat diet on spontaneous tumorigenesis with the effect on carcinogen-induced mutation in or loss of the remaining inherited wild-type* Apc* allele, two of the experimental groups were treated with the food mutagen and carcinogen PhIP. These groups were either exposed to a 45% fat diet both* in utero *and during nursing or a 45% fat diet throughout life. There were no gender differences in the PhIP-treated groups or the control groups without PhIP, and thus the data for males and females are presented together (Figure 9). Both of the dietary groups treated with PhIP showed significantly higher number of small intestinal tumors compared with the untreated groups receiving the same diets (*P* < 0.001 for both comparisons). Hence, PhIP treatment increased intestinal tumorigenesis above the spontaneous level of tumors in the* Min/+* mice, as shown previously [25–27, 33].

In mice treated with PhIP, a 45% fat diet given throughout life gave a significantly higher number of small intestinal tumors compared with exposure to a 45% fat diet during* in utero* and nursing (*P* < 0.001), whereas this effect of diet was not seen in the mice not treated with PhIP.

Small intestinal tumors from mice not treated with PhIP had diameters of 0.2–3.6 mm, whereas the tumor diameters from PhIP-treated mice were 0.2–4.5 mm. The males appeared to have larger tumors than the females (*P* = 0.047 for all mice), however, the difference was not statistically significant in most of the treatment groups, and the direction of this difference was not consistent since in some groups females had larger tumors than males. Therefore, the data are evaluated statistically for females and males together (data not shown).

In the mice exposed to a 45% fat diet* in utero* or during nursing, the differences in tumor diameter were not significantly different compared with the negative control group given a 10% fat diet. In the mice receiving a 45% fat diet during both the* in utero* and nursing periods, the tumors were larger than in the control group (*P* = 0.035), indicating a possible effect on tumor growth from this early exposure to a 45% fat diet. The 45% fat diet during both* in utero* and nursing periods also gave significantly larger tumors compared with all the other groups receiving a 45% fat diet for various periods, including a 45% fat diet as adults and throughout life (*P* < 0.001 for all comparisons). In the mice receiving a 45% fat diet during adult life or throughout life, the tumors were smaller compared with in the negative control mice (*P* < 0.05 for both comparisons), possibly indicating formation of new tumors. The exposure to a 45% fat diet as adults or throughout life gave significantly smaller tumors than in all the other groups exposed to a 45% fat (*P* < 0.05 for all comparisons). The exposure to a 45% fat diet throughout life did not affect the tumor size differently from a 45% fat diet only as adults.

**Figure 10:**
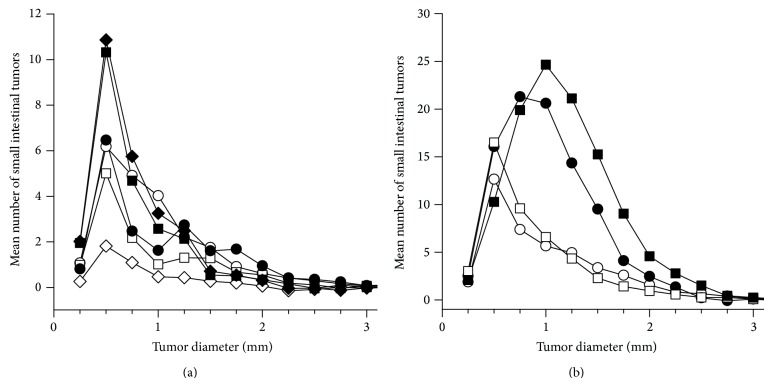
The net effect on tumor size of (a) a 45% fat diet, and (b) of PhIP exposure. (a) The size distribution of small intestinal tumors from untreated pooled female and male* Min/+* mice exposed to a 45% fat diet for various periods is calculated by subtracting the mean number of tumors in the mice exposed to a 10% fat diet throughout life from the mean number of tumors in the treatment groups receiving a 45% fat diet for various periods for each tumor size class. (b) The size distribution of small intestinal tumors from pooled female and male* Min/+* mice exposed to PhIP is calculated by subtracting the mean number of spontaneous tumors in the mice not exposed to PhIP from the mean number of tumors formed in PhIP-treated mice for each tumor size class. The intervals between the tumor size classes are 0.25 mm. The symbols for the experimental groups are as follows: in (a) 10+10+10 (○), 45+10+10 (◊), 10+45+10 (□), 45+45+10 (●), 10+10+45 (◆) 45+45+45 (■), and in (b) the dietary groups without PhIP exposure; 45+45+10 (○), 45+45+45 (□), and the same PhIP-exposed dietary groups with (●) or (■), respectively. The experimental groups are as explained in the legend to Figure 1. *n* = 44–65.

To better illustrate the variations in size of the small intestinal tumors between the dietary groups, curves are shown of the size distribution of tumors (Figure 10(a)), calculated by subtracting the number of tumors in mice exposed to a 10% fat diet throughout life from the number of tumors in the other treatment groups receiving a 45% fat diet for various periods for the different size classes of tumors. It can be seen that the curve for exposure to a 45% fat diet both* in utero* and during nursing is higher than the control group and all other groups to the right, indicating more of the larger tumors, whereas the other treatment groups have curves below the control group for the larger tumor sizes, indicating fewer larger tumors.

PhIP increased the size of the small intestinal tumors compared with the same dietary groups without PhIP (data not shown), both when exposed to a 45% fat diet* in utero* and during nursing (*P* < 0.05) and when exposed throughout life (*P* < 0.05). The PhIP-exposed group given a 45% fat diet throughout life had larger tumors than the PhIP-exposed group given a 45% fat diet* in utero* and during nursing (*P* < 0.05).

The size of the PhIP-induced tumors was illustrated by curves of size distributions of PhIP-induced tumor populations, which were calculated by subtracting the number of tumors in the corresponding dietary group not exposed to PhIP from the number of tumors in the PhIP-treated groups for the difference tumor size classes (Figure 10(b)). It can be seen that the curves for the PhIP-exposed groups are to the right of the curves for the corresponding dietary groups not exposed to PhIP, illustrating larger tumors after PhIP exposure.

### 3.17. Colonic Tumors

Not all of the mice had colonic tumors, regardless of treatment, consistent with results found in our previous experiments with* Min/+* mice [25–27, 33, 34]. There was a higher incidence of colonic tumors in males compared with females in the dietary groups given a 45% fat diet only as adults (*P* = 0.031) and in PhIP-treated mice after exposure during* in utero* and nursing (*P* = 0.030). The only significant difference between the various dietary combinations was a higher incidence of colonic tumors in the female mice exposed to a 45% fat diet only* in utero* versus only as adults (*P* = 0.038). A higher incidence of colonic tumors was observed with PhIP in the group given a 45% fat diet throughout life, in females separately (*P* = 0.016) and in both genders combined (*P* = 0.018), compared with no PhIP-treatment.

The number of colonic tumors was significantly higher in males compared with females for all mice (*P* < 0.001), and in the treatment groups exposed to a 45% fat diet* in utero* and during nursing (*P* = 0.023) and throughout life (*P* = 0.035), hence the data are evaluated separately for females and males (data not shown). Probably because of the very low number of colonic tumors, statistical significance was not reached for any of the differences between the dietary groups. However, except for the comparison with a 45% fat diet* in utero* in females, the number of colonic tumors after the 45% fat diet* in utero* and during nursing was higher than in all the other groups exposed to a 45% fat diet for various periods and not treated with PhIP, in both females and males (data not shown). PhIP significantly increased the number of colonic tumors, in both the dietary group given a 45% fat diet* in utero* and during nursing (*P* = 0.006) and throughout life (*P* = 0.008).

Colonic tumors had diameters of 1.0–5.4 mm in mice from the dietary groups not exposed to PhIP, while the diameter in the PhIP-treated groups ranged from 0.9–6.0 mm. There were no significant differences between the genders or between the various dietary groups in colonic tumor size, and there was no statistically significant effect of PhIP on colonic tumor diameter (data not shown).

### 3.18. Localization of Tumors along the Small Intestine and Colon

**Figure 11:**
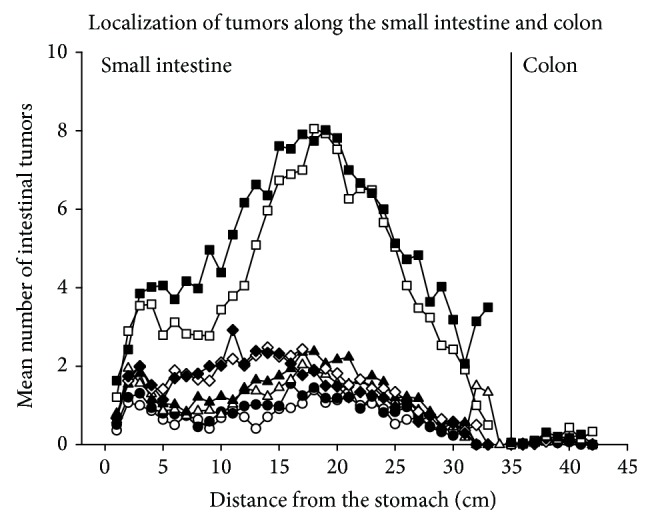
Localization of tumors along the small intestine and colon for pooled female and male* Min/+* mice; 10+10+10 (○), 45+10+10 (●), 10+45+10 (△), 45+45+10 (▲), 10+10+45 (◊) and 45+45+45 (◆), and the PhIP-exposed groups; 45+45+10 (□), and 45+45+45 (■). The tumor position is given as distance from the stomach measured in cm. The experimental groups are as explained in the legend to Figure 1. *n* = 44–65.

The majority of tumors were localized in the distal two-thirds (in the middle and distal parts) of the small intestine in the mice that were exposed to a 10% fat diet throughout life, a 45% fat diet* in utero*, during nursing, or during both* in utero* and nursing (Figure 11), as has usually been found in our previous experiments with* Min/+* mice [26, 27, 33, 34]. The mice that were given a 45% fat diet as adults or throughout life had additional tumors in the proximal part of the small intestine (Figure 11). The PhIP-treated mice had most of the tumors localized in the distal two-thirds of the small intestine and also quite high numbers in the proximal part of the small intestine. This unusual additional increase in tumors proximally in the small intestine was also observed in a previous experiment with genetically-induced obese mice, that is, in* Min/+* mice crossed with* ob/ob* mice, who gets obese when homozygous mutated. Also in the previous experiment as in the present study, the tumor number was increased further in this area of the small intestine when the mice were given a 45% fat diet (another brand in the previous experiment than in the present experiment) (Ngo et al., 2014; unpublished results).

The few colonic tumors present were localized mainly in the middle to distal parts of the colon (Figure 11), as seen in previous experiments with* Min/+* mice [26, 27, 33, 34].

### 3.19. Absolute and Relative Liver and Spleen Weights

To examine if the obesogenic treatment in various periods of life could affect organ weights, in addition to body weight, the absolute and relative weights of liver and spleen were measured at termination of the* Min/+* mice at 11 weeks of age, and of the wild-type mice at 23 weeks of age. In the* Min/+* mice (Table 1), the males had higher absolute liver weight than the females, based on all mice, and in each dietary treatment group separately (*P* < 0.001 for all comparisons). The only significant differences in absolute liver weight found between the dietary treatment groups were that the male mice given a 45% fat diet during both* in utero* and nursing periods had significantly higher absolute liver weight than the mice given a 45% fat diet only as adults (*P* = 0.007) or throughout life (*P* = 0.018). In the* Min/+* males, exposure to a 45% fat diet* in utero* and during nursing without PhIP gave a higher absolute liver weight than the same diet with PhIP exposure (*P* = 0.023). None of these differences between the dietary treatment groups were seen in females.

The relative liver weight in % was not significantly different between the males and females. Compared with the negative control group given a 10% fat diet throughout life, none of the other dietary treatment groups differed in relative liver weight either in female or male* Min/+* mice. In the males, exposure to a 45% fat diet only* in utero*, only during nursing, or* in utero* and during nursing, gave a higher relative liver weight than a 45% fat diet throughout life (*P* < 0.001, *P* = 0.045 and *P* < 0.001, resp.). Exposure to a 45% fat diet throughout life with PhIP gave a higher relative liver weight than the same diet without PhIP exposure in the* Min/+* males (*P* < 0.001) and females (*P* = 0.005). None of the other comparisons of relative liver weight between dietary groups reached significance in the females.

The absolute spleen weight was not significantly different between the* Min/+* females and males. The male* Min/+* mice given a 45% fat diet* in utero* and during nursing had significantly higher absolute spleen weight compared with the negative control mice given a 10% fat diet throughout life (*P* = 0.003), mice given a 45% fat diet only* in utero* (*P* < 0.001), mice given a 45% fat diet as adults (*P* = 0.004) and mice given a 45% fat diet throughout life (*P* = 0.005). In the* Min/+* males, exposure to a 45% fat diet throughout life with PhIP gave a higher absolute spleen weight than the same diet without PhIP exposure (*P* < 0.001). This comparison was also significant in females (*P* = 0.013), whereas none of the other comparisons of absolute spleen weight between the dietary groups reached significance in the females.

The relative spleen weight in % was significantly higher in female than in male* Min/+* mice, based on all mice (*P* = 0.002), but this gender difference did not reach significance in any of the dietary treatment groups separately. The* Min/+* mice given a 45% fat diet* in utero* and during nursing had borderline or significantly higher relative spleen weight compared with the control male mice given a 10% fat diet throughout life (*P* = 0.051), male mice given a 45% fat diet* in utero* (*P* = 0.025), a 45% fat diet as adults (*P* = 0.049 in males and *P* = 0.031 in females), and a 45% fat diet throughout life (*P* = 0.028 in both males and females). The relative spleen weight in* Min/+* mice given a 45% fat diet throughout life with PhIP was significantly higher than the same dietary group without PhIP, in both females and males (*P* < 0.001 for both comparisons).

Also in the wild-type mice (Table 2), the males had higher absolute liver weight than the females, based on all mice (*P* < 0.001), and in each dietary treatment group separately (*P* < 0.001 in all treatment groups, except for the group given a 45% fat diet only during nursing where *P* = 0.019). The only significant differences in absolute liver weight found between the dietary treatment groups in the wild-type mice were that the male mice given a 45% fat diet throughout life had significantly higher absolute liver weight than the control mice given a 10% fat diet throughout life (*P* = 0.028) and the mice given a 45% fat diet only during nursing (*P* = 0.027), whereas these differences were not seen in females. PhIP treatment did not affect the absolute liver weight in the wild-type mice.

The relative liver weight in % was also significantly higher in males than in females, based on all mice (*P* < 0.001), and also in the individual dietary treatment groups of mice given a 45% fat diet* in utero* (*P* = 0.017), as adults (*P* = 0.024), or throughout life (*P* < 0.001). No significant differences were found in relative liver weight between any of the dietary treatment groups in male wild-type mice. In females, the relative liver weight was significantly higher in mice in the negative control group given a 10% fat diet throughout life (*P* < 0.001), a 45% fat diet* in utero* (*P* = 0.003), a 45% fat diet during nursing (*P* < 0.001), or a 45% diet* in utero* and during nursing (*P* < 0.001), compared with the mice given a 45% diet throughout life. In females, the relative liver weight was also significantly higher in mice in the negative control group given a 10% fat diet throughout life (*P* < 0.030), a 45% fat diet during nursing (*P* = 0.008), or a 45% fat diet* in utero* and during nursing (*P* = 0.048), compared with the mice given a 45% diet as adults. PhIP treatment did not affect the relative liver weight in wild-type mice.

There were no significant differences in absolute spleen weight between the genders among the wild-type mice. The only significant difference between the dietary treatment groups was a higher absolute spleen weight in female wild-type mice given a 45% fat diet during nursing compared with as adults (*P* = 0.004), which was not seen in males. PhIP treatment did not affect the absolute spleen weight in wild-type mice.

The relative spleen weight in % was significantly higher in female than in male wild-type mice also, based on all mice (*P* < 0.001), and this difference reached significance in all of the dietary treatment groups separately, except for the group given a 45% fat diet throughout life (*P* values varied from < 0.001 to 0.028). No significant differences were found in relative spleen weight between any of the dietary treatment groups in male mice. Female mice in the negative control group given a 10% fat diet throughout life had significantly higher relative spleen weight compared with the mice given a 45% fat diet as adults, and compared with mice given a 45% fat diet throughout life (*P* = 0.020 for both comparisons). Also female mice given a 45% fat diet only during nursing had significantly higher relative spleen weight compared with the mice given a 45% fat diet as adults, and compared with mice given a 45% fat diet throughout life (*P* < 0.001 for both comparisons). PhIP treatment did not affect the relative spleen weight in wild-type mice.

### 3.20. Serum Leptin Levels

**Figure 12:**
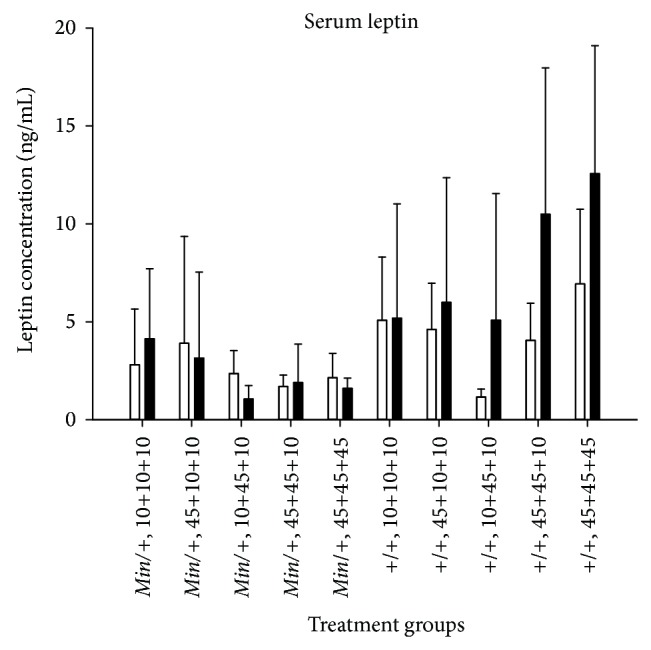
The concentration of leptin in serum at termination was measured in both* Min/+* and wild-type (+/+) mice of both genders from all the dietary groups except 10+10+45 (see explanation in the legend to Figure 1), not treated with PhIP (*n* = 6). Females (white columns), males (black columns).

There were no significant differences in levels of the serum hormone leptin obtained at termination between the genders of mice or between any of the dietary treatment groups (Figure 12). The only significant difference found was that the wild-type mice had significantly higher levels of leptin than the* Min/+* mice. This was found based on all mice (*P* < 0.001), and in the dietary group of mice given a 45% fat diet both* in utero* and during nursing (*P* < 0.001).

## 4. Discussion

The intake of feed per gram body weight in the dams was significantly lower for the 45% fat diet than the 10% fat diet during the second and third week of pregnancy (Figure 2(a)). This was most likely caused by a lower feed intake and/or increased body weight (Figure 4) with the 45% diet, and not by different number of pups per litter, since both dietary groups of dams gave birth to similar mean number of pups per litter.

The mice given a 45% fat diet either as adults (for 8 weeks) or throughout life (for 11 weeks), with or without PhIP, had significantly lower feed intake per gram body weight per week than the other dietary groups receiving a 10% fat diet throughout life (for 11 weeks or 23 weeks), or a 45% fat diet for shorter time, that is, only* in utero* (for 3 weeks), only during nursing (for 3 weeks) or during* in utero* and nursing (for 6 weeks), with or without PhIP (Figure 3). Apparently, there is an adjustment of feed intake, that is, calorie intake, over time, leading to lower intake of the 45% fat diet compared with the 10% fat diet or the 45% fat diet for shorter time. This difference in feed intake between long exposure versus no/short exposure to a 45% fat diet varied with the individual diets and in the individual weeks but reached approximately 20–30% from weeks 5-6 to week 11 in both* Min/+* and wild-type female and male mice, and in most of the weeks in both female and male wild-type mice at weeks 12 to 23. The 45% fat diet has 4.73 kcal/g, whereas the 10% fat diet has 3.85 kcal/g, that is, the 45% fat diet contains 22.9% more kcal per gram diet. Therefore, it appears that the mice adjusted their intake of feed to approximately the same level of kcal, and the effects observed in this experiment are likely caused by the dietary fat as such more than by just the excess calories.

The body weights of the dams during weeks 1 and 2 of pregnancy were significantly higher for the dams on a 45% fat diet compared with dams on a 10% fat diet, although the differences were small, being 3.3% at week 1 and 7.2% at week 2 (Figure 4). At the end of the pregnancy, the difference was only 2.0% and not significant. Since the dams had increased body weight after the 45% fat diet, it is not possible to separate the effect on the offspring* in utero* of increased body weight (obesity) in the dams from the effects of the 45% fat diet as such.

The 10% fat and 45% fat diets both have 20 kcal% protein and the same content of vitamins and minerals. The 10% and 45% fat diets are matched on sucrose, that is, both contain 17% sucrose as percentage of the calories. However, there are differences in other carbohydrates, that is, the 45% fat diet contains less corn starch (291 kcal) and more maltodextrin 10 (a partially hydrolyzed form of corn starch, 400 kcal), than the 10% fat diet (1808.8 kcal corn starch and 300 kcal maltodextrin 10), in total 35 kcal% comes from carbohydrate in the 45% fat diet compared with 70 kcal% in the 10% fat diet. Regarding the content of fat, both diets have the same kcal% from soybean oil (225 kcal), but the 45% fat diet has almost 9 times the content of lard (1598 kcal) as in the 10% fat diet (180 kcal), and in total 45 kcal% comes from fat compared with 10 kcal%.

Since the mice apparently adjust their feed intake over time to approximately the same percentage of calories, the effects observed in this study may be caused by other difference in the diets than calories, that is, by the content of carbohydrates other than sucrose or by the lard (type of fat).

In human studies, various indicators of obesity, such as BMI, waist circumference and waist-to-hip ratio, waist-height-ratio or percentage of body fat, have been found to be more or less strongly associated with study end points [36]. In this work, we have evaluated obesity in three different ways; as body weight development using AUC calculated for a specified time period, as body weight at a specific age and as terminal BMI.

When evaluated as AUC from age 3-4 days to 11 weeks, there was an obesogenic effect of an early exposure to a 45% fat diet* in utero* and during nursing, or only during nursing, showing that the intrauterine and nursing period is a susceptible window of exposure to a high fat diet for later development of obesity as adults (Figures 6(a)–6(d)). However, this effect in AUC from day 3-4 to week 11 of an early exposure to a 45% fat diet was no longer present when evaluated as AUC for the age of 12 to 23 weeks in the wild-type mice (Figures 6(e) and 6(f)), indicating that this effect was transient. Similarly, the observed effects of an early exposure to a 45% fat diet during* in utero* and nursing, or only during nursing, on body weight at age 11 weeks were no longer present when the wild-type mice had reached the age of 23 weeks. As opposed to body weight development as AUC from day 3-4 to week 11 and body weight at 11 weeks, the end point terminal BMI at 11 weeks did not demonstrate the sensitive period for obesity early in life from exposure to a 45% fat diet in the* Min/+* mice. Likewise, the end point terminal BMI at 23 weeks did not demonstrate in the wild-type mice the sensitive period for obesity early in life from exposure to a 45% fat diet. So for this end point, BMI was not as sensitive as body weight at a specific age, and even less sensitive, compared with AUC for body weight development. AUC, which integrates the changes in body weight over a longer period of time, was the best end point in this experiment. BMI was also found to be the least sensitive and specific of several obesity-related predictors of metabolic syndrome in a human population [36].

In addition to affecting the body weight of the mice, the early exposure to a 45% fat diet during both* in utero* and nursing periods increased the organ weights compared with the much longer exposure to a 45% fat diet later in life, that is, as adults or throughout life. This was observed for the relative liver weight compared with a 45% fat diet throughout life in male* Min/+* mice (Table 1), and compared with a 45% fat diet as adults or throughout life in female wild-type mice (Table 2). Also the relative spleen weight after exposure to a 45% fat diet during both* in utero* and nursing periods was increased compared with a 45% fat diet as adults or throughout life in both female and male* Min/+* mice (Table 1). The absolute liver weight and the absolute spleen weight were also increased by the exposure to a 45% fat diet during both* in utero* and nursing periods compared with a 45% fat diet as adults or throughout life in male* Min/+* mice (Table 1).

Maternal obesity and developmental programming of metabolic disorders in the offspring have been studied extensively in animal models [37–40]. Animal studies indicate that both the fetal period and the postnatal period may be critical windows for development, and hence, alterations in the nutrition during these periods could induce metabolic programming effects, which may be manifested as pathological conditions later in life. Several physiological and metabolic mechanisms are not fully matured at birth and continue maturation in the immediate postnatal period, for example, neurons and pancreatic islets continue to develop after birth in rodents [41]. Many studies have exposed female animals for a high fat diet early in life; during pregnancy or during lactation, or during both periods, and found long-term consequences of metabolic and endocrine pathophysiology in one or both genders of the offspring as adults, both in mice [42–44] and rats [45–48]. Interestingly, prenatal stress seems to have similar effects as a high fat diet for increased susceptibility to diet-induced obesity in the offspring [47]. Relevant data also come from human studies. For instance, it was shown that high pregnancy weight gain was associated with increased body weight of the offspring in childhood, and that this effect was only partially mediated through higher birth weight [49]. However, this question is still not resolved in humans, since in another study, maternal overweight/obesity was associated with early deceleration of growth, seen as less weight gain, less length growth and less fat mass at three months of age [50]. A systematic review of maternal and paternal body mass index and offspring obesity concluded that there was only limited evidence to support the fetal overnutrition hypothesis in humans [51].

However, we have compared the outcome of exposure to a 45% fat diet among several specific periods of life. For body weight measured as AUC from day 3-4 to week 11 and as body weight at age 11 weeks, and for the number of small intestinal tumors, exposure only* in utero* had less effect that exposure during nursing, or during both the* in utero* and nursing periods. The diameter of small intestinal tumors was also increased after exposure during both the* in utero* and nursing periods. These results point to exposure via the milk during the nursing period as more important than exposure* in utero* for these effects. Another study in mice found that a high fat diet limited to the lactation period caused diet-induced obesity in the male offspring [44]. Also, a study in rats showed that maternal high fat diet during the nursing period had greater influence on the offspring's metabolic phenotype and body weight than prenatal (*in utero*) high fat diet [52]. For obvious reasons, studies in humans are not able to compare obesogenic exposure in separated periods of life, such as during pregnancy and the lactation period. However, in a case-cohort study of Danish children, it was found that infant weight and weight gain already during the first months of life were associated with obesity in childhood [53]. They did not observe any particular critical time period during infancy related to the weight gain from 2 weeks to 9 months of age; odds ratios increased from 1.27 associated with an increase of 1 weight-tertile from age 2 weeks to 1 month, and to 1.54 from 2 to 3 months of age, thereafter the odds ratio were stable until 9 months of age.

Our data stress the importance of early exposure to obesogenic conditions compared with the same exposure later in life, that is, as adults. For instance, the exposure to a 45% fat diet as adults did not increase body weight as AUC from day 3-4 to week 11 compared with the control group or any of the other exposure groups, whereas the exposure to a 45% fat diet during nursing actually gave a significantly higher AUC than exposure as adults* in Min/+* male mice (*P* = 0.011). Also for body weight of* Min/+* and wild-type mice at 11 weeks of age and terminal BMI for* Min/+* mice at 11 weeks of age, the effects of a 45% fat diet as adults did not increase the values compared with exposure* in utero*, during nursing, or during both* in utero* and nursing. One could speculate if this effect was only due to differences in length of exposure to a 45% fat diet, rather that exposure in a particular period. However, this is not the case, since exposure as adults is from weaning at age 3 weeks until age 11 weeks for* Min/+* mice, and from weaning at age 3 weeks until age 23 weeks for wild-type mice, that is, in 8 and 20 weeks, respectively. Exposure throughout life is from conception to termination at 11 weeks for* Min/+* mice, and from conception to termination at 23 weeks for wild-type mice, that is, 14 and 26 weeks, respectively. In comparison, the exposure to a 45% fat diet* in utero* was for 3 weeks, the exposure during nursing was for 3 weeks, and the exposure both* in utero* and during nursing was then for 6 weeks, all much shorter than the exposures to a 45% fat diet as adults and throughout life.

As opposed to the situation at 11 weeks, when comparing body weight after exposure as adults with the earlier exposure periods using AUC for body weight, body weight at 23 weeks or terminal BMI at 23 weeks in wild-type mice, the adult exposures increase these parameters compared with exposure* in utero*, during nursing, or both* in utero* and during nursing, indicating that the importance of early exposure has disappeared some time between 11 and 23 weeks of age. This early exposure effect seen at 11 weeks, but not at 23 weeks, is not caused by a difference between* Min/+* mice (terminated at 11 weeks) and wild-type mice (terminated at 23 weeks), since both genotypes are implicated in these results at 11 weeks.

Maternal obesity may have consequences for production and secretion of adipokines from the adipose tissues, such as leptin. Leptin has been shown to be important for placental function and maternal-fetal exchange processes regulating growth and development, and in later stages of pregnancy central leptin resistance occurs to allow increased nutrient availability for the fetus [54]. Disruption of signaling capacity of leptin associated with obesity is a potential risk factor leading to pregnancy complications as a result of fuel partitioning* in utero*.

The only significant difference in serum leptin levels measured at termination in this study was that the* Min/+* mice had significantly lower levels of leptin than the wild-type mice (Figure 12). At the same time, the body weight as AUC from day 3-4 to week 11 (Figure 6) and the body weight at week 11 were lower in* Min/+* mice (Figure 5(a)) than in wild-type mice (Figure 5(b)). Therefore, in this experiment higher leptin levels were associated with increased body weight (in wild-type mice), whereas lower levels of leptin were associated with lower body weight (in* Min/+* mice). The wild-type mice results are more similar to the situation in humans where obesity is associated with elevated levels of leptin, representing a form of leptin resistance [55, 56], as opposed to the* ob/ob* mouse model where the lack of leptin is associated with extreme obesity (see [30, 31], Ngo et al., 2014; unpublished results).

Alterations in nutrition, such as feeding a high fat diet, during critical periods of prenatal or postnatal development may induce permanent changes in the responsiveness of hypothalamic neurons to hormone signals regulating energy homeostasis and feeding, leading to increase in food intake, preference for fatty food, hyperlipidemia and higher body weight, thereby affecting the chances of becoming obese later in life [57–59]. Leptin and insulin are likely hormonal mediators for the environmental nutrient sensing system that controls feeding. Data implicate a postnatal leptin surge to be an important trophic factor for the development of hypothalamic feeding circuits and is critical for normal energy balance and hypothalamic regulation of feeding later in life [59]. Data also show that changes in insulin levels, specifically hyperinsulinemia, during pregnancy could induce alterations in hypothalamic organization that may affect metabolism of the offspring later in life [59].

The* Min/+* mice (Figure 7(a)) had significantly higher nonfasted blood glucose levels than the wild-type mice (Figure 7(b)) at 6 weeks (*P* < 0.001), and also a larger AUC in GTT after being fasted (Figure 8). Since the feed intake in this experiment was recorded per litter, which consisted of both* Min/+* and wild-type mice, it is not known whether there was a difference in feed intake between* Min/+* and wild-type mice that could explain their differences in blood glucose levels. However, this is not likely, since our previous experiment found similar feed intake in* Min/+* and wild-type mice measured in metabolic cages (Ngo et al., 2014; unpublished results). Also, the body weight was lower in* Min/+* mice than in wild-type mice for body weight evaluated as AUC from day 3-4 to 11 weeks (Figure 6) and as body weight at 11 weeks (Figure 5). However, an alternative explanation for the difference in blood glucose levels may be that APC is involved in regulation of epithelial glucose transport in the intestines, since* Min/+* mice had increased activity of the electrogenic glucose carrier (SGLT1) compared with wild-type mice [60]. APC is a component of the Wnt signaling pathway [19, 20]. Other intriguing possibilities of a relationship between APC and blood glucose levels come from data showing that the Wnt signaling pathway, which is as an important modulator of adipocyte differentiation [61, 62], also influences endocrine pancreas development and modulates mature *β*-cell functions, including insulin secretion, survival and proliferation, and thereby may be involved in the pathogenesis of diabetes [63]. Components of the Wnt signaling pathway may also be involved in determining susceptibility to diet-induced obesity [64].

Based on all values from* Min/+* mice at 6 and 11 weeks, the PhIP-treated mice had higher blood glucose levels than mice not treated with PhIP (*P* = 0.004) (Figure 7(a)), which was also observed previously (Ngo et al., 2014; unpublished results), whereas this effect of PhIP was not significant for the wild-type mice at weeks 6 and 23 (Figure 7(b)). Blood glucose AUC in GTT was also higher in PhIP-exposed mice compared with mice not given PhIP after exposure to a 45% fat diet throughout life (*P* < 0.001), but not after exposure to a 45% fat diet* in utero* and during nursing (Figure 8). Therefore, at one or several conditions favoring intestinal tumorigenesis, that is,* Min/+* mutation, exposure to the carcinogen PhIP and a 45% fat diet, the blood glucose levels were increased.

Exposure to the 45% fat diet only* in utero* did not significantly increase the number of spontaneous small intestinal tumors compared with the negative control group given a 10% fat diet throughout life (Figure 9). Exposure to a 45% fat diet only during the nursing period was significantly increased compared with the negative control group (*P* < 0.05), and exposure to a 45% fat diet during both the* in utero* and nursing periods significantly increased the tumor numbers further compared both with the negative control group (*P* < 0.05), and the 45% fat diet only* in utero* (*P* < 0.05), but not compared with only during nursing. Thus, the effect of a 45% fat diet during the nursing period is more efficient in increasing the tumor number than the exposure* in utero*. These data show a direct adverse effect on the offspring as adults from obesogenic conditions early in life. Also, if comparing the tumor results using litter instead of individual mice as the statistical unit, the main findings are essentially similar. The exceptions are that the tumor number after a 45% fat diet only during nursing was no longer significantly higher compared with a 10% fat diet throughout life, and a 45% fat diet throughout life was no longer significantly higher than a 45% fat diet* in utero* or only during nursing (data not shown).

Both exposures to a 45% fat diet in adult life or throughout life gave a significant increase in small intestinal tumors compared with the negative control group, the group given a 45% fat diet* in utero* and the group given a 45% fat diet only during nursing (*P* < 0.05 for all comparisons). Exposure to a 45% fat diet in adult life or throughout life did not significantly increase the number of small intestinal tumors compared with the early exposure during both* in utero* and nursing, indicating the importance of early life exposure for intestinal tumor development by a high fat diet. However, it is a discrepancy in these results, since it would be expected that exposure to a 45% fat diet throughout the whole life should give tumor numbers approaching the sum of tumor numbers after exposure to a 45% fat diet* in utero* and during nursing (i.e., early in life) and as adults (the rest of the life), whereas the tumor numbers in these three groups were not significantly different, that is, they were similar.

It is not known whether a 45% fat diet in these mice had an effect on tumor initiation, by causing mutation or loss of heterozygosity (LOH) in the remaining wild-type allele of the* Apc* gene, or affected promotion, that is, the growth of already initiated stem cells in the intestines, causing more tumors to reach a size detectable in the microscope at termination, or if both mechanisms were involved. These factors will affect the final tumor numbers measured at a specific time point. Lipotoxic free fatty acids from the 45% fat diet [65–67] and/or the subsequent obesity [68] may cause oxidative stress and accumulation of reactive oxygen species (ROS), which may have led to subsequent DNA damage and tumor initiation in the intestines.

Regarding exposure to a 45% fat diet early versus late in life, the effects on body weight coincide well with the effects on small intestinal tumor number, implicating an association between obesity and intestinal tumorigenesis. There was an obesogenic effect of an early exposure to a 45% fat diet* in utero* and during nursing, or only during nursing, when evaluated as AUC from age 3-4 days to 11 weeks or as body weight at 11 weeks, and there was significantly increased number of tumors after early exposure to a 45% fat diet during nursing or during both the* in utero* and nursing periods. The exposure to a 45% fat diet as adults was not able to increase body weight as AUC from day 3-4 to 11 weeks, body weight at 11 weeks or BMI at 11 weeks compared with the early exposure during both* in utero* and nursing, and similarly, a 45% fat diet as adults was not able to increase the tumor number compared with the early exposure during both* in utero* and nursing.

In mice treated with PhIP, a 45% fat diet given throughout life gave a significantly higher number of small intestinal tumors compared with exposure to a 45% fat diet during* in utero* and nursing (*P* < 0.001), whereas this dietary effect was not seen in the mice not treated with PhIP. In the PhIP-exposed* Min/+* mice there were no differences in either AUC from day 3-4 to 11 weeks, body weight at 11 weeks or terminal BMI at 11 weeks between exposure to a 45% fat diet during* in utero* and nursing, or throughout life. This is difficult to interpret since PhIP itself decreased the body weight evaluated with all three body weight parameters, probably because of the increased tumor burden. Possibly, the weight-decreasing effect of PhIP could have counteracted the weight-increasing effect of the 45% fat diet, or the fat itself more than the body weight affected the number of PhIP-induced small intestinal tumors. In an earlier experiment, a 45% fat diet during adult life did not increase the number of PhIP-induced small intestinal tumors compared with exposure to a 10% fat diet in* Min/+ × ob* mice (Ngo et al., 2014; unpublished results).

The 45% fat diet during both* in utero* and nursing periods also gave significantly larger tumors compared with the negative control group and all the other groups receiving a 45% fat diet for various periods, including a 45% fat diet as adults and throughout life (*P* < 0.001 for all comparisons), indicating a possible effect on tumor growth from this early exposure to a 45% fat diet (Figure 10(a)). The opposite was seen with exposure to a 45% fat diet later in life. The exposure to a 45% fat diet as adults or throughout life gave significantly smaller tumors than in the negative control mice and all the other groups exposed to a 45% fat diet early in life (*P* < 0.05 for all comparisons), possibly indicating formation of new tumors. The exposure to a 45% fat diet throughout life did not affect the tumor size differently from a 45% fat diet only as adults.

The PhIP-induced tumors were both increased in number and significantly larger compared with the spontaneous tumors in the small intestine (Figure 10(b)). Whether the larger diameter of these tumors was due to an earlier induction, a faster growth, or a combination of both events, is not known.

The mice that were given a 45% fat diet as adults or throughout life had additional tumors in the proximal part of the small intestine, which was also seen after PhIP exposure (Figure 11). This unusual increase in tumors proximally in the small intestine, in addition to the more common distribution in the middle and distal parts of the small intestine, was also observed in a previous experiment with genetically-induced obese mice, that is, in* Min/+* mice crossed with* ob* mice, who gets obese when homozygous mutated (Ngo et al., 2014; unpublished results).

In the previous experiment, the number of proximal tumors was especially high in the obese* ob/ob* mice and was further increased with another brand of a 45% fat diet. The reason for this phenomenon therefore seems to be associated with genetical or diet-induced obesity. It could be speculated whether it is caused by the increased secretion of bile acid into this small intestinal area caused by the high fat diet, as had been suggested to cause intestinal cancer [69].

In this experiment, blood glucose levels were measured and GTT was performed to study the hypothesis of disrupted blood glucose regulation as a link between obesity and intestinal tumorigenesis [28, 29]. However, based on both end points a shorter exposure to a 45% fat diet early in life, that is,* in utero*, during nursing, or both* in utero* and during nursing, was apparently not able to affect the blood glucose levels, whereas a longer exposure to a 45% fat diet as adults or throughout life did increase blood glucose. Therefore, in this experiment the exposure time to a 45% fat diet during the early periods of life was too short to affect the glucose levels, and the effects of the 45% fat diet and obesity on the intestinal tumorigenesis worked through a mechanism independent of blood glucose regulation. However, it should be noted that the only significant difference between later and early exposure was that exposure to a 45% diet as adults, and not throughout life, increased blood glucose levels compared with exposure both* in utero* and during nursing at 6 and 23 weeks in female wild-type mice separately (*P* = 0.018).

It is suggested that prenatal genetic or environmental factors may disrupt early development and produce long-term increased susceptibility of the offspring to new metabolic challenges later in life, causing various pathological conditions. However, in this study, we have shown that the exposure to obesogenic conditions early in life directly affected obesity in adults on a 10% fat diet without the need for a challenge in the form of exposure to a 45% fat diet also as adults. Similarly, the number of tumors formed spontaneously because of the inherited mutation in* Apc* was increases after early exposure to obesogenic conditions without a second dietary challenge as adults.

There is growing interest also in the developmental origins of cancer [70–72]. Also in this context, there are proposed influences of adipokines, such as leptin and adiponectin [73], and also insulin, on central mechanisms of energy balance, causing irreversible changes in hypothalamic neural interconnections leading to obesity and cancer. Another possibility is epigenetic modifications of specific genes, including tumor suppressor genes or oncogenes, and their altered expression, leading to cancer development. Not only a high fat diet, but other nutritional components may impact on developmental origin of cancer, since it was shown in rats that dietary protein type (soy protein isolate versus casein) during pregnancy had different effects on azoxymethane-induced colon tumor number and colon tissue gene expression, as well as serum IGF-I and testosterone levels in the offspring as adults [74]. It has also been shown in humans that body fatness at ages 5 and 10 years and higher adult height were associated with increased risk of distal adenoma, but not proximal or rectal adenoma, later in life, independent of adult body weight [75].

Apparently, the effect on adult body weight of exposures early in life disappeared sometime between 11 and 23 weeks of age. In spite of this observation, it would still be interesting to see if the effects on both body weight and intestinal tumorigenesis were transferred to the next generations, and if so, what would be the mechanisms for this transgenerational transfer. Even if the effects should be transient, they are present into reproductive age, and as such could affect the subsequent offspring.

It may be that the worldwide epidemic of obesity and subsequent risk of diabetes or metabolic syndrome and other illnesses may not only be a result of our own lifestyle of inadequate activity and poor diet as adults, but it may also be propagated and increased earlier in life because of unhealthy metabolic conditions* in utero*, that is, with fetal overgrowth/adiposity or undergrowth, as well as by obesogenic conditions during early childhood. Prevention, rather than treatment, is of interest.

There seems to be an opportunity to potentially break the cycle of obesity during pregnancy leading to obese children [76], if managing to make obese women lose weight and achieve a normal body weight/BMI prior to conception. It has been shown that maternal obesity increased both maternal and placental inflammation, but that this inflammation could be reduced by an increase in N-3/N-6 fatty acid ratio, which limited the adverse effects on the child of the developmental programming caused by the maternal obesity [77]. Therefore, even if obese mothers are not able to lose weight before or during pregnancy, improving their nutrition during pregnancy may affect the outcome of the metabolic disturbances on the fetus.

## 5. Conclusions

We have demonstrated in* Min/+* mice that the intrauterine and nursing period is a window of susceptibility for exposure to a high fat diet for development of obesity, and also that this obesogenic exposure has direct adverse health effects, that is, increased intestinal tumorigenesis, in the offspring as adults. Disturbed blood glucose regulation does not appear to be involved in this association between obesity and intestinal tumorigenesis.

## Figures and Tables

**Table 1:** Effects of exposure to a 45% fat diet during various periods of life on absolute liver weight (ALW), relative liver weight (RLW), absolute spleen weight (ASW), and relative spleen weight (RSW) in female and male *Min/+* mice terminated at 11 weeks of age (mean of individual mice in the group ± SD).

Experimental group	*n*	BW (g)	ALW (g)	RLW (%)	ASW (g)	RSW (%)
*Min*/+, females						
10 + 10 + 10	22	19.4 ± 1.6	0.74 ± 0.18	3.81 ± 0.74	0.12 ± 0.03	0.65 ± 0.21
45 + 10 + 10	17	20.1 ± 1.8	0.82 ± 0.16	4.08 ± 0.58	0.12 ± 0.05	0.60 ± 0.22
10 + 45 + 10	27	21.4 ± 1.8	0.82 ± 0.11	3.84 ± 0.55	0.15 ± 0.09	0.69 ± 0.42
45 + 45 + 10	18	20.1 ± 1.7	0.82 ± 0.10	4.10 ± 0.46	0.17 ± 0.11	0.86 ± 0.60^a,b^
10 + 10 + 45	34	20.9 ± 2.6	0.75 ± 0.13	3.64 ± 0.52	0.12 ± 0.05	0.58 ± 0.22^a^
45 + 45 + 45	22	23.2 ± 2.6	0.84 ± 0.14	3.66 ± 0.47^a^	0.13 ± 0.05^a^	0.55 ± 0.22^b,c^
45 + 45 + 10 PhIP	22	20.6 ± 2.2	0.86 ± 0.10	4.23 ± 0.54	0.21 ± 0.11	1.00 ± 0.55
45 + 45 + 45 PhIP	15	19.5 ± 2.9	0.83 ± 0.14	4.35 ± 0.84^a^	0.23 ± 0.11^a^	1.23 ± 0.68^c^
*Min*/+, males						
10 + 10 + 10	17	27.4 ± 2.6	1.10 ± 0.19	4.01 ± 0.55	0.12 ± 0.05^a^	0.45 ± 0.21^a^
45 + 10 + 10	29	27.8 ± 2.6	1.16 ± 0.16	4.16 ± 0.36^a^	0.13 ± 0.07^b^	0.46 ± 0.26^b^
10 + 45 + 10	30	28.3 ± 2.8	1.14 ± 0.21	3.99 ± 0.55^b^	0.16 ± 0.08	0.59 ± 0.31
45 + 45 + 10	19	28.8 ± 2.2	1.22 ± 0.21^a,b,c^	4.23 ± 0.67^c^	0.21 ± 0.13^a,b,c,d^	0.75 ± 0.47^a,b,c,d^
10 + 10 + 45	25	28.0 ± 3.2	1.05 ± 0.15^a^	3.78 ± 0.56	0.13 ± 0.06^c^	0.48 ± 0.20^c^
45 + 45 + 45	29	29.9 ± 3.2	1.07 ± 0.15^b^	3.58 ± 0.45^a,b,c,d^	0.14 ± 0.06^d,e^	0.47 ± 0.22^d,e^
45 + 45 + 10 PhIP	27	25.3 ± 3.0	1.09 ± 0.14^c^	4.34 ± 0.49	0.23 ± 0.11	0.94 ± 0.52
45 + 45 + 45 PhIP	29	26.5 ± 5.6	1.09 ± 0.20	4.20 ± 0.84^d^	0.26 ± 0.09^e^	1.00 ± 0.39^e^

Relative liver weight (RLW) (%) = absolute liver weight (ALW)/body weight (BW) × 100, relative spleen weight (RSW) (%) = absolute spleen weight (ASW)/BW × 100. g = gram.

^a–e^Dietary treatment groups within each gender with similar letters are significantly different.

**Table 2:** Effects of exposure to a 45% fat diet during various periods of life on absolute liver weight (ALW), relative liver weight (RLW), absolute spleen weight (ASW), and relative spleen weight (RSW) in female and male wild-type mice terminated at 23 weeks of age (mean of individual mice in the group ± SD).

Experimental group	*n *	BW (g)	ALW (g)	RLW (%)	ASW (g)	RSW (%)
+/+, females						
10 + 10 + 10	26	25.9 ± 3.3	1.03 ± 0.34	3.91 ± 0.87^a,e^	0.12 ± 0.03	0.45 ± 0.13^a,b^
45 + 10 + 10	24	26.8 ± 4.2	1.03 ± 0.32	3.79 ± 0.85^b^	0.11 ± 0.03	0.41 ± 0.12
10 + 45 + 10	21	28.0 ± 3.9	1.15 ± 0.38	4.04 ± 0.89^c,f^	0.14 ± 0.09^a^	0.50 ± 0.37^c,d^
45 + 45 + 10	31	27.6 ± 3.9	1.08 ± 0.35	3.84 ± 0.83^d,g^	0.11 ± 0.03	0.42 ± 0.14
10 + 10 + 45	26	31.3 ± 6.2	1.02 ± 0.22	3.27 ± 0.49^e,f,g^	0.10 ± 0.02^a^	0.33 ± 0.10^a,c^
45 + 45 + 45	31	34.3 ± 4.0	1.04 ± 0.23	3.02 ± 0.51^a,b,c,d^	0.11 ± 0.02	0.34 ± 0.09^b,d^
45 + 45 + 10 PhIP	21	27.0 ± 3.6	1.11 ± 0.34	4.06 ± 0.87	0.12 ± 0.03	0.43 ± 0.13
45 + 45 + 45 PhIP	21	33.9 ± 7.5	1.05 ± 0.32	3.09 ± 0.66	0.11 ± 0.02	0.34 ± 0.11
+/+, males						
10 + 10 + 10	25	35.0 ± 5.1	1.43 ± 0.41^a^	4.02 ± 0.75	0.11 ± 0.02	0.34 ± 0.11
45 + 10 + 10	36	36.0 ± 4.3	1.54 ± 0.26	4.28 ± 0.53	0.11 ± 0.02	0.30 ± 0.07
10 + 45 + 10	24	37.2 ± 4.2	1.42 ± 0.24^b^	3.81 ± 0.47	0.11 ± 0.02	0.30 ± 0.07
45 + 45 + 10	27	36.4 ± 5.6	1.51 ± 0.37	4.18 ± 0.90	0.10 ± 0.03	0.29 ± 0.09
10 + 10 + 45	27	40.5 ± 7.5	1.54 ± 0.53	3.75 ± 0.83	0.10 ± 0.02	0.25 ± 0.07
45 + 45 + 45	46	42.9 ± 5.8	1.73 ± 0.59^a,b^	3.95 ± 0.98	0.12 ± 0.02	0.28 ± 0.08
45 + 45 + 10 PhIP	25	38.5 ± 4.1	1.49 ± 0.30	3.84 ± 0.47	0.12 ± 0.04	0.32 ± 0.15
45 + 45 + 45 PhIP	28	44.6 ± 5.0	1.70 ± 0.47	3.76 ± 0.74	0.11 ± 0.02	0.24 ± 0.05

Relative liver weight (RLW) (%) = absolute liver weight (ALW)/body weight (BW) × 100, relative spleen weight (RSW) (%) = absolute spleen weight (ASW)/BW × 100. g = gram.

^a–f^Dietary treatment groups within each gender with similar letters are significantly different.
